# Learning physics-consistent particle interactions

**DOI:** 10.1093/pnasnexus/pgac264

**Published:** 2022-11-18

**Authors:** Zhichao Han, David S Kammer, Olga Fink

**Affiliations:** Institute for Building Materials, ETH Zürich, 8093 Zürich, Switzerland; Institute for Building Materials, ETH Zürich, 8093 Zürich, Switzerland; Laboratory of Intelligent Maintenance and Operations Systems, EPFL, 1015 Lausanne, Switzerland

**Keywords:** interacting particle systems, pairwise interaction, graph neural networks, deterministic physics operator, physics consistency

## Abstract

Interacting particle systems play a key role in science and engineering. Access to the governing particle interaction law is fundamental for a complete understanding of such systems. However, the inherent system complexity keeps the particle interaction hidden in many cases. Machine learning methods have the potential to learn the behavior of interacting particle systems by combining experiments with data analysis methods. However, most existing algorithms focus on learning the kinetics at the particle level. Learning pairwise interaction, e.g., pairwise force or pairwise potential energy, remains an open challenge. Here, we propose an algorithm that adapts the Graph Networks framework, which contains an edge part to learn the pairwise interaction and a node part to model the dynamics at particle level. Different from existing approaches that use neural networks in both parts, we design a deterministic operator in the node part that allows to precisely infer the pairwise interactions that are consistent with underlying physical laws by only being trained to predict the particle acceleration. We test the proposed methodology on multiple datasets and demonstrate that it achieves superior performance in inferring correctly the pairwise interactions while also being consistent with the underlying physics on all the datasets. While the previously proposed approaches are able to be applied as simulators, they fail to infer physically consistent particle interactions that satisfy Newton’s laws. Moreover, the proposed physics-induced graph network for particle interaction also outperforms the other baseline models in terms of generalization ability to larger systems and robustness to significant levels of noise. The developed methodology can support a better understanding and discovery of the underlying particle interaction laws, and hence, guide the design of materials with targeted properties.

Significance Statement Understanding, modeling, and predicting the kinetic behavior of interacting particle systems rely on knowing the governing interaction laws between individual particles. However, in real applications, the ground truth information on pairwise interactions remains unknown. Here, we propose a Graph Neural Network framework that incorporates universal physical laws to infer pairwise force (or potential energy) for any particle system. The proposed method precisely infers pairwise particle interactions that are consistent with underlying physical laws without any supervision by only being trained to predict particle acceleration. The proposed methodology is a step forward in developing flexible and robust tools for the discovery of physical laws, which will be the basis for various applications such as designing new materials.

## Introduction

Interacting particle systems play a key role in nature and engineering as they govern planetary motion (1), mass movement processes (2) such as landslides and debris flow, bulk material packaging (3), magnetic particle transport for biomaterials (4), and many more. Since the macroscopic behavior of such particle systems is the result of interactions between individual particles, knowing the governing interaction law is required to better understand, model, and predict the kinetic behavior of these systems. Particle interactions are determined by a combination of various factors including contact, friction, electrostatic charge, gravity, and chemical interaction, which affect the particles at various scales. The inherent complexity of particle systems inhibits the study of the underlying interaction law. Hence, they remain largely unknown and particle systems are mostly studied in a stochastic framework or with simulations based on simplistic laws.

Recent efforts on developing machine learning (ML) methods for the discovery of particle interaction laws have shown great potential in overcoming these challenges (5–11). These ML methods, such as the *Graph Network-based Simulators* (GNS) (12) for simulating physical processes, *Dynamics Extraction From cryo-em Map* (DEFMap) (13) for learning atomic fluctuations in proteins, the *SchNet* (14,15) which can learn the molecular energy, and the *neural relational inference model* (NRI) (16) developed for inferring heterogeneous interactions, can be applied on various types of interacting particle systems such as water particles, sand, and plastically deformable particles. They allow implicit and explicit learning of the mechanical behavior of particle systems without prior assumptions and simplifications of the underlying mechanisms. A commonly applied approach is to predict directly the kinetics of the particles without explicitly modeling the interactions (12, 14,17–21). The neural networks, then, map directly the input states to the particle acceleration, occasionally by virtue of macroscopic potential energy (12, 14, 17–19). While these approaches give an accurate prediction of the particle system as it evolves, they do neither provide any knowledge about the fundamental laws governing the particle interactions nor are they able to extract the particle interactions precisely.

Recent work (22) proposed an explicit model for the topology of particle systems, which imposes a strong inductive bias and, hence, provides access to the individual pairwise particle interactions. Reference (22) demonstrated that their Graph Network (GN) framework predicts well the kinetics of the particles system. However, as we will show, the inferred particle interactions violate Newtonian laws of motion, such as the action–reaction property, which states that two particles exert the same but opposed force onto each other. Therefore, the extracted pairwise particle interactions do not correspond to the real underlying particle interaction *force* or *potential*, which are the fundamental properties of a physical system. The origin of these discrepancies lies in the design of the GN approach, which does not sufficiently constrain the output space, and clearly demonstrates the need for a physics-consistent Graph Neural Network (GNN) framework for particle interactions.

Besides interacting particle systems, recent works applied GN models to learn microlevel dynamics in other physical systems. For example, the work of Moon et al. (23) integrated domain knowledge into the GNN to learn drug–target binding affinities in a supervised way. Ha and Jeong (24) trained the GN model as a simulator for interacting discrete physical systems other than interacting particles, including cellular automata, the Vicsek model, the active Ornstein–Uhlenbeck particle model, and the movement of a flock of birds. Another follow-up research line aimed to improve GNNs to learn particle dynamics in more complicated scenarios. For example, the work of Huang et al. (25) learns particle dynamics in interacting particle systems with geometrical constraints where some particles are connected by bounds. However, to the best of our knowledge, no other previous work aimed to reveal physics-consistent particle interactions from observed particles trajectories without any direct supervision.

**Figure 1:**
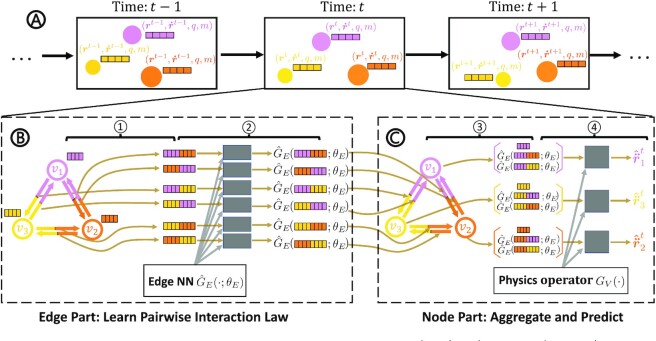
Framework of the proposed model to learn pairwise force or pairwise potential energy. (A) The interacting particle system contains three particles that evolve over time. At every time step, each particle is described by multiple features, which include position, velocity, charge, and mass (represented by the bar). Position and velocity evolve with time whereas other properties remain constant. (B to C) The proposed method learns physics-consistent pairwise force or pairwise potential at every time step *t*. The model has two components: the edge part (B) and the node part (C). In the edge part (B), two nodes’ vectors are concatenated as edge feature (process ①). An edge neural network $\hat{G}_E(\cdot ; \theta _{E})$ (*θ_E_* represents the trainable parameters) takes the edge feature as input (process ②) and outputs a learnt vector on that edge representing the pairwise force or potential energy. In the node part (C), the output vectors by the edge neural network and the raw node feature are aggregated on each node (process ③). We design the deterministic node operator *G_V_*( · ) by incorporating physics knowledge to derive the net acceleration on nodes (process ④). By minimizing the loss on node-level accelerations, the edge neural network $\hat{G}_E(\cdot ; \theta _{E})$ will output pairwise force or potential energy exactly.

Here, we propose a GNN framework that incorporates universal physical laws, specifically Newton’s second law of motion, to learn the interaction potential and force of any physical particle system. The proposed algorithm, termed physics-induced graph network for particle interaction (PIG’N’PI), combines the GNN methodology with deterministic physics operators to guarantee physics consistency when indirectly inferring the particle interaction forces and potential energy (Fig. 1). We will show that PIG’N’PI learns the pairwise particle potential and force by only being trained to predict the particle acceleration (without providing any supervision on the particle interactions). Moreover, we will show that the inferred interactions by PIG’N’PI are physically consistent (contrary to those inferred by purely data-driven approaches). We will further demonstrate that predictions provided by PIG’N’PI are more accurate, generalize better to larger systems, and are more robust to noise than those provided by purely data-driven graph network approaches. Moreover, we will demonstrate on a case study that is close to real applications that the proposed algorithm is scalable to large systems and is applicable to any type of particle interaction laws.

## PIG’N’PI: physics-induced graph network for particle interaction

We propose a framework that is able to infer pairwise particle forces or potential energy by simply observing particle motion in time and space. In order to provide physics-consistent results, a key requirement is that the learnt particle interactions need to satisfy Newtonian dynamics. One of the main challenges in developing such a learning algorithm is that only information on the particle position in time and space along with particle properties (e.g., charge and mass) can be used for training the algorithm and no ground truth information on the interactions is available since it is very difficult to measure it in real systems.

The proposed framework comprises the following distinctive elements (Fig. 1): (1) a graph network with a strong inductive bias representing the particles, their properties, and their pairwise interactions; and (2) physics-consistent kinetics imposed by a combination of a neural network for learning the edge function and a deterministic physics operator for computing the node function within the graph network architecture. In addition, the proposed framework consists of the two steps: (1) training the network to predict the particle motion in time and space; and (2) extraction of the pairwise forces or the pairwise potential energy from the edge functions of the trained network.

*Particle systems*: We consider particle systems that are moving in space and time and are subject to Newtonian dynamics without any external forces. A particle system in this research is represented by the directed graph *G* = (*V, E*), where nodes *V* = {*v*_1_, *v*_2_, …, *v*_|*V*|_} correspond to the particles and the directed edges *E* = {*e_ij_*: *v_i_*, *v_j_* ∈ *V, i* ≠ *j*} correspond to their interactions. The graph is fully connected if all particles interact with each other. Each particle *i*, represented by a node *v_i_*, is characterized by its time-invariant properties, such as charge *q_i_* and mass *m_i_*, and time-dependent properties such as its position $\boldsymbol {r}_i^t$ and its velocity $\boldsymbol {\dot{r}}_i^t$. We use $\boldsymbol {\eta }_i^t$ to denote the features of particle *i* at time *t*, $\boldsymbol {\eta }_i^t = [\boldsymbol {r}_i^t, \boldsymbol {\dot{r}}_i^t, q_i, m_i]$. We limit our evaluations to particle systems comprising homogeneous particle types. This results in particles exhibiting only one type of interaction with all its neighboring particles, leading to |*E*| = |*V*|(|*V*| − 1). We further assume that the position $\boldsymbol {r}_i^t$ of each particle *i* is observed at each time step *t* and that this information is available during training. Based on the position information $\boldsymbol {r}_i^t$, velocity $\boldsymbol {\dot{r}}_i^t$, and acceleration $\boldsymbol {\ddot{r}}_i^t$ are computed.

**Figure 2:**
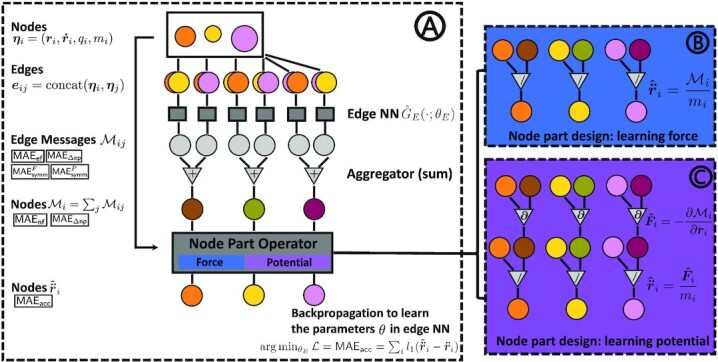
PIG’N’PI. (A) The workflow where the edge neural network $\hat{G}_E(\cdot ; \theta _{E})$ takes edge features as input. The corresponding output message $\mathcal {M}_{ij}$ is the predicted pairwise force or potential energy, depending on the physics operator (B) or (C) in the node part. Parameters *θ_E_* in $\hat{G}_E(\cdot ; \theta _{E})$ are trained by minimizing the loss on particle acceleration.

*Proposed framework*: The proposed PIG’N’PI framework extends the general GN framework proposed by (26), which is a generalized form of message-passing GNNs. The architecture of the proposed framework is illustrated in Fig. 2. We use a directed graph to represent the interacting particle system where nodes correspond to the particles and edges correspond to their interactions. The framework imposes a strong inductive bias and enables to learn the position-invariant interactions across the entire particle system network. Given the particle graph structure, the input is then defined by the node features $\boldsymbol {\eta }_i$ (representing particle’s characteristics). The target output is the acceleration $\boldsymbol {\ddot{r}}_i^{t}$ of each node at time step *t*. The standard GNs block (26), typically, comprises two neural networks: an edge neural network $\hat{G}_E(\cdot ; \theta _{E})$ and a node neural network $\hat{G}_V(\cdot ; \theta _{V})$, where *θ_E_* and *θ_V_* are the trainable parameters. Here, we propose to substitute the node neural network $\hat{G}_V(\cdot ; \theta _{V})$ by a deterministic node operator *G_V_*( · ) to ensure that the learned particle interactions are consistent with the underlying physical laws. The main novelty compared to the standard GN framework is that we impose known basic physical laws to ensure that the inferred pairwise force or potential energy corresponds to the real force or potential energy while only being trained on predicting the acceleration of the particles. Whether to use the force-based PIG’N’PI (blue color in Fig. 2) or the potential-based PIG’N’PI (purple color in Fig. 2) depends on different demands or downstream applications in practice, i.e., we can use force-based PIG’N’PI if we are only interested in the pairwise force without caring about the pairwise potential energy; or we can use potential-based PIG’N’PI if we want to know the pairwise potential. Furthermore, note that the force-based PIG’N’PI method is not limited to energy-conserving systems, but can also be extended to dissipative systems (e.g., friction).

It is important to emphasize that only information on particle positions is used for training the algorithm and the ground-truth information on the forces and the potential energy is not available during training. For each edge, the property vectors $\boldsymbol {\eta }_i$ of two nodes connected by an edge are concatenated as the edge feature vector. The edge neural network $\hat{G}_E(\cdot ; \theta _{E})$ outputs a message on every edge that corresponds to the pairwise force or potential energy. The output dimension is set to be the same as the spatial dimension *d* (two or three) if $\hat{G}_E(\cdot ; \theta _{E})$ is targeted to learn the pairwise force or one to learn the pairwise potential energy. Edge messages are aggregated on nodes and the node part operator computes the output corresponding to the acceleration of nodes, imposing physics-consistency on edge messages. Trained to predict the node-level acceleration, once applied to a new particle system, the GN predicts the particle motion at consecutive time steps. The pairwise forces or the pairwise potential energy can then be extracted from the edge function for each time step.

*Contributions of the present work compared to precious research*: Here, we propose a methodology to learn the *pairwise* force or *pairwise* potential energy from the observed particle trajectories. This focus distinguishes our work from many previous works such as (14,27–31) that learn the energy of the system and then derive the *per-particle* dynamics from the global energy. Moreover, as outlined above, our proposed approach does not have access to any ground truth information during training but rather learns to infer the force and potential energy indirectly. This is contrary to the previously proposed approaches that rely on such information (17, 21).

While our proposed framework has several similarities with two previously proposed frameworks that are also aiming to infer pairwise force and pairwise potential energy using also only the particle accelerations for training (22), none of the previously proposed methods is able to infer the underlying particle interactions that are consistent with the underlying physical laws. We demonstrate in our experiments that the learnt particle interactions of the previously proposed approaches are not consistent with the underlying physical laws and do not correspond to the real forces or potential energy.

In fact, the proposed algorithm has also similarities to the Physics-informed neural networks (PINNs) (32), which aim to solve partial differential equations. Both PINNs and PIG’N’PI integrate known physical laws. While PIG’N’PI integrates Newton’s second law, PINNs enforce the structure imposed by partial differential equation at a finite set of collocation points.

## Results and discussion

### Performance evaluation metrics

We evaluate the performance of the proposed PIG’N’PI framework on synthetic data generated from two- (*d* = 2) and three- (*d* = 3) dimensional numerical simulations. The key distinctive property of the generated datasets is the definition of the inter-particle potential energy *P*, which defines the inter-particle pairwise force by $\boldsymbol {F}= -\partial P/ \partial \boldsymbol {r}$. The selected cases, which have also been used in prior work (22) and can be considered as a benchmark case study, cover a wide range of particle interaction features, including dependence on particle properties, e.g., mass and charge, dependence on interaction properties, e.g., stiffness, and varying degrees of smoothness (see Table 1 and Fig. S2 in SI Appendix J for visualization).

**Table 1.** The force and potential energy equations for different datasets, where $\boldsymbol {F}_{ij}$ is the force from particle *j* to particle *i, P_ij_* is the potential incurred by particle *j* on particle *i, r_ij_* is the Euclidean distance between particle *i* and particle *j*, $\boldsymbol {n}_{ij}$ is the unit vector pointing from particle *i* to particle *j*, and *q_i_* and *m_i_* are the electric charge and mass of particle *i. k, L, c*, and Θ are constants.

**Dataset**	**Pairwise force** ($\boldsymbol {F}_{ij}$)	**Pairwise potential (*P_ij_*)**
Spring	$k(r_{ij}- L)\boldsymbol {n}_{ij}$	$\frac{1}{2}k(r_{ij}- L )^2$
Charge	$-cq_i q_j\boldsymbol {n}_{ij}/r_{ij}^2$	*cq_i_q_j_*/*r_ij_*
Orbital	$m_i m_j\boldsymbol {n}_{ij}/r_{ij}$	*m_i_m_j_*ln(*r_ij_*)
Discnt	$\boldsymbol {0}$ , if *r_ij_* < Θ	0, if *r_ij_* < Θ
	$(r_{ij} -1)\boldsymbol {n}_{ij}$ , otherwise	0.5(*r_ij_* − 1)^2^, otherwise

The method developed by ref. (22), which applies multilayer perceptrons [MLPs (33)] in the edge and node part, serves as the baseline for comparison. We do not change the architecture of the baseline except for changing the output dimension of its edge part MLPs when learning the pairwise force or potential energy. The output dimension is a *d*-dimensional vector for learning the pairwise force and a one-dimensional scalar for the potential energy. Besides the baseline, we compare the performance of PIG’N’PI to an alternative method proposed by ref. (34) that is also based on GN and was specifically designed to infer pairwise forces. We denote this method as GN+ and the details are introduced in the “Details of the method to learn pairwise force introduced by (34)” section.

We split each dataset into training, validation, and testing datasets and use $\mathcal {T}_{\text{train}}$, $\mathcal {T}_{\text{valid}}$, and $\mathcal {T}_{\text{test}}$ to indicate the corresponding simulation time steps for these different splits. Details regarding the dataset generation are provided in the “Details about simulation and experiments” section. The baseline algorithm and PIG’N’PI are trained and evaluated on the same training and testing datasets from simulations with an 8-particle system. Further, the evaluation of the generalization ability uses a 12-particle system.

It should be noted that ref. (22) measures the quality of the learnt forces by quantifying the linear correlation between each dimension of the learnt edge message and all dimensions of the ground-truth pairwise force. This is a necessary but not sufficient condition to claim the correspondence of the learnt edge message with the pairwise interactions and to evaluate the performance of the indirect inference of the pairwise interactions. Instead, we evaluate the proposed methodology with a focus on two key aspects: (1) supervised learning performance, and (2) consistency with underlying physics. For all the evaluations, the mean absolute error on the testing dataset of various particle and interaction properties is used and is defined as follows

(1) $$\begin{eqnarray*}
\textsf {MAE}^{\mathrm{inter}} (\hat{\phi }, \phi ) = \frac{1}{|\mathcal {T}_{\text{test}}|} \frac{1}{|E|} \sum _{t\in \mathcal {T}_{\text{test}}}\sum _{i,j}^{i\ne j} l_1(\hat{\phi }_{ij}^t,\phi _{ij}^t) ~,
\end{eqnarray*}
$$

and

(2) $$\begin{eqnarray*}
\textsf {MAE}^{\mathrm{part}} (\hat{\phi }, \phi ) = \frac{1}{|\mathcal {T}_{\text{test}}|} \frac{1}{|V|} \sum _{t \in \mathcal {T}_{\text{test}}} \sum _{i=1}^{|V|} l_1(\hat{\phi }_i^t,\phi _i^t) ,
\end{eqnarray*}
$$

respectively, where the superscript hat indicates the predicted values. Here, $\hat{\phi }_{ij}$ and *ϕ_ij_* are the predicted and corresponding ground-truth, respectively, of a physical quantity between particle *j* and particle *i* (e.g., pairwise force), and $\hat{\phi }_{i} = \sum _j \hat{\phi }_{ij}$ and ϕ_*i*_ = ∑_*j*_ϕ_*ij*_ are the aggregated prediction and the corresponding ground-truth, respectively, on particle *i* (e.g., net force). *l*_1_(*x, y*) computes the sum of absolute differences between each element in *x* and *y, l*_1_(*x, y*) = ∑_*i*_|*x_i_* − *y_i_*|, if *x* and *y* are vectors or the absolute difference, *l*_1_(*x, y*) = |*x* − *y*|, if *x* and *y* are scalars. Hence, $\textsf {MAE}^{\mathrm{part}}$ measures the averaged error of the physical quantity on particles over $\mathcal {T}_{\text{test}}$, and $\textsf {MAE}^{\mathrm{inter}}$ is the averaged error of the inter-particle physical quantity over $\mathcal {T}_{\text{test}}$.

The supervised learning performance is evaluated on the prediction of the acceleration MAE_acc_$= \textsf {MAE}^{\mathrm{part}}(\boldsymbol {\hat{\ddot{r}}},\boldsymbol {\ddot{r}})$. The true acceleration values serve as target values during training. The physical consistency is evaluated on two criteria. First, we evaluate the ability of the proposed framework to infer the underlying physical quantities that were not used as target during training (e.g., pairwise force), and second, we evaluate physical consistency by verifying whether Newton’s action–reaction property is satisfied.

The following metrics are used to evaluate the consistency with the true pairwise interaction. For pairwise force, we use MAE_ef_$= \textsf {MAE}^{\mathrm{inter}}(\boldsymbol {\hat{F}},\boldsymbol {F})$; and for potential energy case, we evaluate the increment in potential energy MAE_Δep_$= \textsf {MAE}^\mathrm{inter} (\hat{P}-\hat{P}^0, P-P^0)$, where superscript 0 refers to the initial configuration.

For the second part of the evaluation of the physical consistency, we verify whether Newton’s action–reaction property is satisfied. For that, we evaluate the symmetry in either inter-particle forces with $\textsf {MAE}_\textsf {symm}^{F}= \frac{1}{|\mathcal {T}_{\text{test}}|} \frac{1}{|E|} \sum _{t\in \mathcal {T}_{\text{test}}}\sum _{i,j}^{i\ne j} l_1(\boldsymbol {\hat{F}}_{ij}^t, - \boldsymbol {\hat{F}}_{ji}^t)$ or inter-particle potential with $\textsf {MAE}_\textsf {symm}^{P}= \frac{1}{|\mathcal {T}_{\text{test}}|} \frac{1}{|E|} \sum _{t\in \mathcal {T}_{\text{test}}}\sum _{i,j}^{i\ne j} l_1(\hat{P}_{ij}^t, \hat{P}_{ji}^t)$.

### Performance evaluation between PIG’N’PI, GN+, and baseline for pairwise force

**Figure 3:**
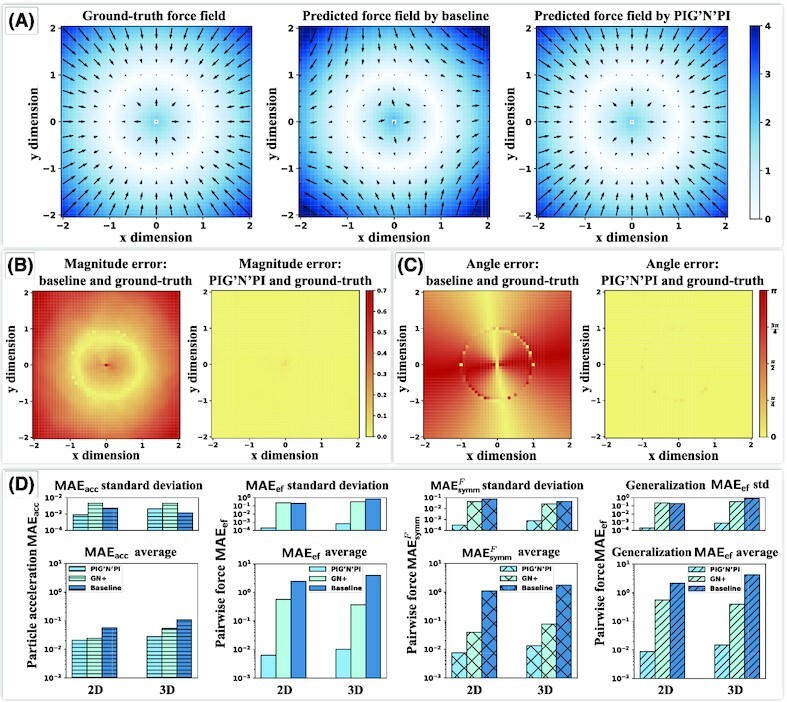
Case study: quality of pairwise force prediction of PIG’N’PI and the baseline model on two-dimensional spring dataset. (A) The spring force field around a given particle. Color indicates the force amplitude. From left to right: ground-truth spring force field, predicted force field by the baseline model, predicted force field by PIG’N’PI. (B) The magnitude error between predicted force and the ground-truth force ($\vert \text{norm}(\boldsymbol {\hat{F}})-\text{norm}(\boldsymbol {F}) \vert$). Left is the result of baseline model and right is the result of PIG’N’PI. (C) The angle difference between predicted force and the ground-truth force ($\text{Angle}(\boldsymbol {\hat{F}}, \boldsymbol {F})$, in radian). Left is the result of baseline model and right is the result of PIG’N’PI. (D) Comparison of the quality of PIG’N’PI, GN+, and baseline model on learning the pairwise force, where bottom is average result of five experiments and top is the corresponding standard deviation. From left to right (in logarithmic scale): acceleration error MAE_acc_, pairwise force error MAE_ef_, force symmetry error $\textsf {MAE}_\textsf {symm}^{F}$, and pairwise force error MAE_ef_ on generalization dataset.

First, we analyze PIG’N’PI for application on particle systems with interactions given by pairwise forces. We start with evaluating the supervised learning performance by evaluating the prediction of the acceleration using MAE_acc_. The results show that PIG’N’PI provides slightly better predictions than the GN+ and the baseline model for both the spring dataset (see Fig. 3D) and all other datasets (see SI Appendix B).

**Figure 4:**
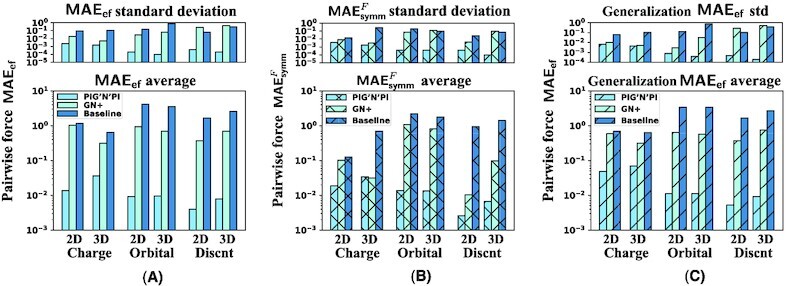
Quality of pairwise force prediction of PIG’N’PI, the GN+, and the baseline model. We report the average and standard deviation of different errors with five experiments, in logarithmic scale. (A) Pairwise force prediction error MAE_ef_. (B) Pairwise force symmetry error $\textsf {MAE}_\textsf {symm}^{F}$. (C) Pairwise force error MAE_ef_ on generalization dataset.

To verify the physical consistency, we first evaluate if the implicitly inferred pairwise forces are consistent with the true physical quantity. PIG’N’PI is able to infer the force field around a particle correctly, while the baseline model fails to predict the force field (see Fig. 3A for the spring dataset). A force field needs to be precise in both amplitude and direction. The error of the magnitude (see Fig. 3B) and angle (see Fig. 3C) demonstrate unambiguously the superior performance of PIG’N’PI compared to the baseline model. We quantitatively summarize the performance of the pairwise force inference with MAE_ef_, which shows PIG’N’PI is able to infer the pairwise force correctly, while both GN+ and the baseline fail to infer the pairwise force [two to three orders of magnitude worse inference performance for the spring dataset (see Fig. 3D) and all other datasets (see Fig. 4A and SI Appendix B)].

Secondly, we verify the consistency of the implicitly inferred pairwise forces with Newton’s action–reaction law by evaluating the symmetry of the inter-particle forces with $\textsf {MAE}_\textsf {symm}^{F}$. Our results demonstrate that PIG’N’PI satisfies the symmetry property. However, GN+ and the baseline model are not able to satisfy the underlying Newton’s laws for the spring dataset (see Fig. 3D) and the other datasets (see Fig. 4B and SI Appendix B).

Furthermore, we test the robustness of PIG’N’PI and the baseline model to learn from noisy data. We impose noise to the measured positions and then compute the noisy velocities (first-order derivative of position) and noisy accelerations (second-order derivative of position). The noisy accelerations serve then as the target values for the learning tasks of all the models. The performance of PIG’N’PI decreases with increasing noise level (see SI Appendix I; Tables S11, S12, and S13). This is to be expected given that adding noise makes the training target (particle accelerations) less similar to the uncorrupted target that is associated with particle interactions. However, PIG’N’PI can still learn reasonably well the particle interactions despite the corrupted data. The performance of the baseline model fluctuates; however, with different noise levels significantly. This is due to the fact that the baseline model does not learn the particle interactions but rather the particle kinematics and is, therefore, more sensitive to noise.

Finally, we note that the proposed algorithm is also able to generalize well when trained on an 8-particle system and applied to a 12-particle system for all datasets (see Figs. 3D and 4, and Table S4).

Overall, the results demonstrate that the proposed algorithm learns correctly the pairwise force (that is consistent with the underlying physics) without any direct supervision, i.e., without access to the pairwise force in the first place, and that the inferred forces are consistent with the imposed underlying physical laws.

### Performance evaluation between PIG’N’PI and baseline for pairwise potential energy

**Figure 5:**
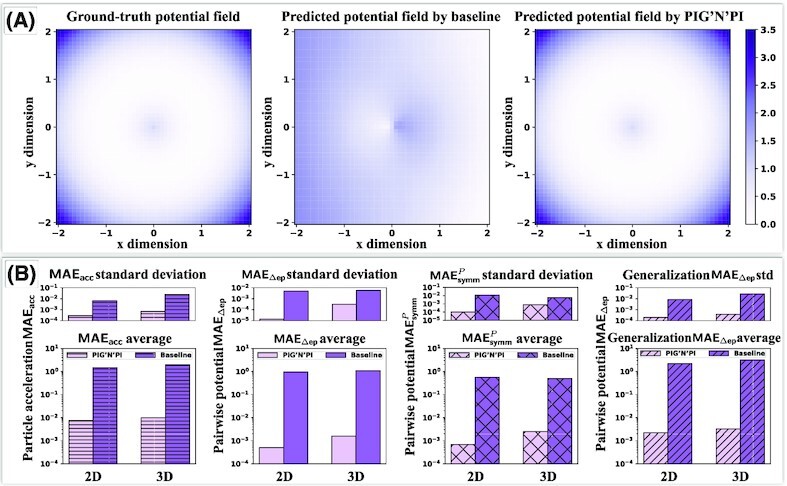
Case study: quality of pairwise potential prediction of PIG’N’PI and the baseline model on Spring dataset. (A) The spring potential field around a given particle. Color indicates the potential amplitude. From left to right: ground-truth spring potential field, predicted potential field by the baseline model, predicted potential field by PIG’N’PI. (B) Comparison of the quality of PIG’N’PI and baseline model on learning the pairwise force. From left to right (in logarithmic scale): acceleration error MAE_acc_, pairwise potential error MAE_Δep_, potential symmetry error $\textsf {MAE}_\textsf {symm}^{P}$, and pairwise potential error MAE_Δep_ on generalization dataset.

Besides learning the pairwise force, the proposed methodology is extended to learn the pairwise potential energy (see node part design for learning potential in the “PIG’N’PI: physics-induced graph network for particle interaction” section). In this case, the physics operator computes the pairwise force via partial derivative. Since GN+ was solely designed for learning the pairwise force, it is not possible to apply it to infer the pairwise potential. Therefore, for the task of pairwise potential energy inference, we only compare PIG’N’PI with the baseline model. Our results show that PIG’N’PI performs well in the supervised learning of the acceleration (MAE_acc_). Here again, its performance is considerably better compared to the baseline model (see Fig. 5B). Moreover, the performance is similar to that in the force-based version of the algorithm.

However, when comparing the performance of the baseline model on the supervised learning task between the potential-based version and the force-based version of the model, the performance reduces significantly in the potential-based implementation (compare to Fig. 3D). This drop of performance is potentially explained by the adjustment of the output dimension of the edge neural network in the baseline model to enable the extraction of the potential energy.

**Figure 6:**
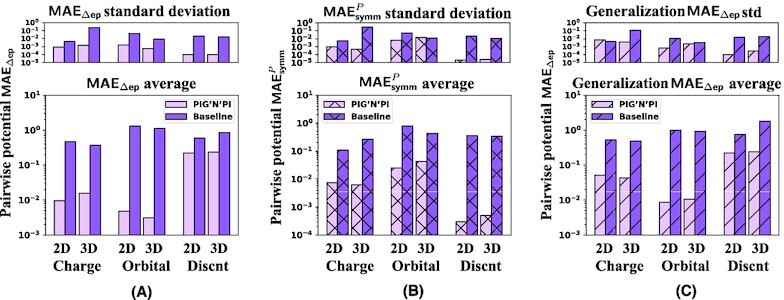
Quality of pairwise potential prediction of PIG’N’PI and the baseline model. We report different errors in terms of consistency with the underlying physical laws, in logarithmic scale. (A) Pairwise potential incremental error MAE_Δep_. (B) Pairwise potential symmetry error $\textsf {MAE}_\textsf {symm}^{P}$. (C) Pairwise potential error incremental error MAE_Δep_ on generalization dataset.

Further, our results demonstrate a superior performance of the PIG’N’PI algorithm on consistency with underlying physics. Firstly, it infers well the increment of the potential energy (see Fig. 5A). It clearly infers the inrement of the potential field correctly. On the contrary, the baseline model fails to infer the potential energy. This can be quantitatively assessed with MAE_Δep_. The results (see Figs. 5B and 6) show that PIG’N’PI is able to infer the potential energy, while the performance of the baseline model indicates that it is not able to learn the potential energy only from observing particles movements.

It is important to note that our algorithm cannot predict the absolute value of the potential energy; only the increment (see SI Appendix D; Fig. S1). The reason is that the model is trained on the acceleration, which is computed from the derivative of the potential energy (i.e., the force). Hence, the model only constrains the derivative, and the constant of integration remains unknown. This limitation can be overcome by one of the two following options: either the potential energy is constrained by a spatial boundary condition or by an initial condition. In the former, we can impose a known value for a given value of *r_ij_*, e.g., we could use the assumption that the potential energy for a charge interaction approaches zero with increasing particle distance. The alternative (but less likely) approach consists in knowing the potential energy at a given time, e.g., at the beginning of the observation and add the inferred increment to the known initial value. Nevertheless, knowing the absolute value of the potential energy is, in fact, not crucial as only its derivative determines the dynamics of a particle system. This is also confirmed in our experiments by the accurate prediction of the acceleration (see MAE_acc_).

Secondly, similar to the pairwise force prediction, PIG’N’PI also provides a superior performance on satisfying Newton’s action–reaction property, while again, the baseline model fails to satisfy the underlying Newton’s law. The performance is quantified by the symmetry of the inter-particle potential energies ($\textsf {MAE}_\textsf {symm}^{P}$). (Figs. 5B and 6, and Table S3).

Finally, we test the generalization ability of the learning algorithms in a similar way as in the pairwise force case study. We apply the models trained on an 8-particles system to a new particle system comprising 12 particles. The results (see SI Appendix C; Table S5) show that PIG’N’PI predicts well the pairwise force and potential energy, and outperforms considerably the baseline model (see Figs. 5B and 6). This demonstrates that the PIG’N’PI model provides a general model for learning particle interactions.

### Case study under more realistic conditions: learning pairwise interactions for an Lennard-Jones (LJ)-argon system

To evaluate the performance of the proposed framework on a more realistic system with a larger particle interaction system (to evaluate the scalability), we apply PIG’N’PI on a large LJ system.

We adopt the dataset introduced in ref. (35). This dataset simulates the movements of liguid argon atoms governed by the LJ potential. The LJ potential, which is given by *V*(*r*) = 4ϵ{(σ/*r*)^1^2 − (σ/*r*)^6^}, is an extensively used governing law for two nonbonding atoms (36). The simulation contains 258 particles in a cubic box whose length is 27.27 Å. The simulation is run at 100 K with periodic boundary conditions. The potential well depth ϵ is set to 0.238 kilocalorie/mole, the van der Waals radius σ is set to 3.4 Å, and the interaction cutoff radius for the argon atoms is set to 3σ. The mass of argon atom is 39.9 dalton. The dataset is run for 10 independent simulations. Each simulation contains 1000 time steps with randomly initialized positions and velocities. The position, velocity, and acceleration of all particles are recorded at each time step.

**Figure 7:**
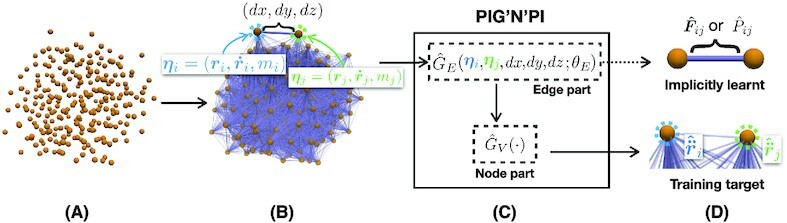
Pipeline of PIG’N’PI to learn pairwise force or potential for the LJ-argon particle data. Solid-line arrows indicate the data processing path from input to the output. The dash-line arrow depicts the intermediate output of every edge corresponding to the inferred pairwise force or potential energy. (A) Positions of 258 particles at a random time step. (B) Representation of the constructed graph. Node features comprise position, velocity, and mass. Edge features comprise the relative position difference under periodic boundary conditions. (C) PIG’N’PI. Edge part takes the concatenation of two nodes’ features and the edge feature as input and infers the pairwise force or potential. Node part aggregates the output on every edge and predicts the acceleration. (D) The inferred pairwise force or potential by edge part and the acceleration by node part.

Figure 7 summarizes the learning pipeline. Contrary to the previous case study where for a small number of particles, a fully connected graph is considered, in this case study, we construct the graph of neighboring particles at every time step (Fig. 7). We connect the particles within the defined interaction cutoff radius while taking the periodic boundary conditions into consideration. Particles in the LJ-argon system are characterized by their position, velocity, and mass. The charge is not part of the particle properties, which is different from the particle systems considered in the previous case study (section “Performance evaluation between PIG’N’PI, GN+, and base-line for pairwise force” and section “Performance evaluation between PIG’N’PI and baseline for pairise potential energy”). Moreover, we compute the position difference under the periodic boundary condition and use it as an edge feature. This edge feature is required because the distance between two particles in this simulation does not correspond to the Euclidean distance in the real world due to the periodic boundary conditions. The node features and the edge features are then concatenated and are used as the input to the edge part of PIG’N’PI (Fig. 7C) or the baseline model. Similarly to the previous case study, the learning target is the accelerations of the particles. The pairwise force and pairwise potential energy are then inferred from the intermediate output of edge part.

**Figure 8:**
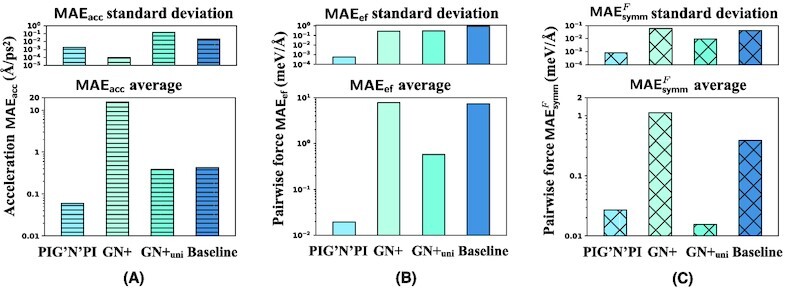
Performance of the algorithms on pairwise force predictions on the LJ-argon system. We report the MAE on the acceleration prediction, which is the target for the learning task (A), the MAE on the pairwise force inference (indirect inference task) MAE_ef_ (B), and the consistency with Newton’s action–reaction property: the MAE on pairwise force symmetry $\textsf {MAE}_\textsf {symm}^{F}$ (C). The average (plots at the bottom) on logarithmic scale and standard deviation (plots in the top row) are computed from five experiments.

We evaluate the performance of PIG’N’PI on inferring pairwise interactions of the LJ-argon particle system with the same performance metrics as in the previous case study. The results are reported in Fig. 8 and Table S6. Because the particles in this dataset have the same mass, we also test a variant of GN+ such that we assign all nodes with a unique learnable scalar. We denote this variant as GN+_uni_. The results confirm the very good performance of PIG’N’PI as observed in the previous case study. Generally, GN+_uni_ outperforms the GN+, but PIG’N’PI still surpasses GN+_uni_ and the baseline. On the one hand, PIG’N’PI performs better than the baseline, GN+, and GN+_uni_ on the supervised prediction task of predicting the acceleration [achieving less than half of the MAE_acc_ compared to the baseline and the GN+_uni_ (Fig. 8A)]. On the other hand, PIG’N’PI is also able to infer the learn pairwise force correctly. Again, the baseline model is not able to infer the pairwise force (PIG’N’PI outperforms the baseline by more than two orders of the magnitude on the MAE_ef_). Moreover, particle interactions inferred by PIG’N’PI are consistent with Newton’s action–reaction law ($\textsf {MAE}_\textsf {symm}^{F}$). The bad performance of the baseline model indicates that the learnt interactions do not satisfy Newton’s law.

To summarize, similar to the cases discussed in the “Performance evaluation between PIG’N’PI, GN+, and base-line for pairwise force” section, PIG’N’PI learns the pairwise force well without any direct supervision for this complex and large system.

**Figure 9:**
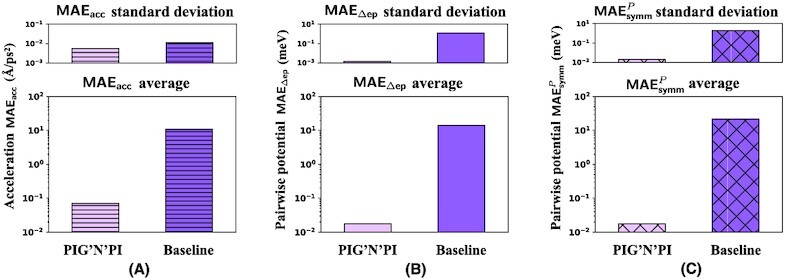
Quality of pairwise potential prediction on the LJ-argon data. We report different errors in logarithmic scale. The average and standard deviation are computed from five experiments. (A) Acceleration prediction error MAE_acc_. (B) Pairwise potential incremental error MAE_Δep_. (C) Pairwise potential symmetry error $\textsf {MAE}_\textsf {symm}^{P}$.

Besides, we test PIG’N’PI to learn the pairwise potential energy for this LJ system. Results are reported in Fig. 9 and Table S7. We first examine the MAE_acc_ that is the learning target. The MAE_acc_ of PIG’N’PI is similar to that in the force-based version of the algorithm. PIG’N’PI performs significantly better than the baseline model with more than two orders of magnitude (Fig. 9A). And, similar to the cases in the “Performance evaluation between PIG’N’PI and baseline for pairwise potential energy” section, we again observe the performance drop of the baseline model in this potential-based version with the force-based version. Then, we evaluate the MAE_Δep_ and MAE_ef_ that are the two metrics for measuring the quality of the learnt pairwise potential energy. Results show that PIG’N’PI provides again superior performance on inferring the increment of the potential energy MAE_Δep_ (Fig. 9B). The bad performance of the baseline model clearly shows that it is not able to infer the potential energy correctly. Finally, we check Newton’s action–reaction property in the potential energy by $\textsf {MAE}_\textsf {symm}^{P}$. Here again, the potential energy inferred by PIG’N’PI follows Newton’s laws while the baseline model fails to infer the underlying interaction laws correctly (Fig. 9D). All evaluations demonstrate that the predicted pairwise potential energy by PIG’N’PI is consistent with the LJ potential used in the simulation, even though PIG’N’PI does not access the ground truth information.

The results on this case study demonstrate the scalability of PIG’N’PI to larger systems and the applicability to more realistic case studies. Moreover, the results confirm the results obtained in the previous case study that PIG’N’PI is able to infer the underlying interaction laws correctly. While the other baseline methods are able to precisely predict the particle dynamics, they fail to infer the underlying pairwise interaction laws correctly.

### Comparison of PIG’N’PI to alternative hyperparameter choices and an alternative regularized architecture

We compare the performance of the proposed approach first to alternative hyperparameter choices, in particular to different activation functions, and second, to an alternative way of imposing physical consistency in the network architecture.

First, we evaluate different choices of activation functions following the observations made in previous studies (18, 21, 37) that confirmed their significant influence on the performance of MLPs in approximating physical quantities. The performance of PIG’N’PI with different activation functions is reported in Table S8 in SI Appendix F and Table S9 in SI Appendix G. The results demonstrate that PIG’N’PI with SiLU activation function (which was in fact used in all case studies) performs consistently best on most test datasets compared to PIG’N’PI with other commonly used activation functions, such as ReLU or LeakyReLU. Based on this observation, the performance of the baseline with the SiLU activation function was evaluated (SI Appendix B). The results show that the SiLU activation improves the learning performance of the baseline model to some degree (when only evaluating the prediction performance MAE_acc_). However, it still performs consistently worse than PIG’N’PI and, more importantly, the consistency with underlying physics in terms of the inferred force (or potential) and interaction symmetry worsens even con-siderably.

Second, we compare the performance of PIG’N’PI to an alternative way of imposing physical consistency: we add a regularization into the baseline model to enforce the symmetry property onto the output messages of the edge function. The goal of imposing the symmetry regularization term is to ensure that the model satisfies the action–reaction physical consistency requirement. It is expected that by satisfying this symmetry constraint, the model performance on learning physics-consistent pairwise forces and potential energy can be improved. We add a symmetry regularization term on the learnt pairwise corresponding messages to enforce the action–reaction property. The details on this regularization term can be found in the “Details on imposing a symmetry regularization on the base-line model” section.

While the performance is improved compared to the baseline model without any regularization, the results demonstrate that PIG’N’PI still performs considerably better on inferring physical meaningful quantities for the pairwise force and potential energy than the symmetry-regularized baseline model (see SI Appendix H; Table S10 for detailed results and SI Appendix E for the evaluation on the LJ-argon system).

## Conclusions

In this paper, we propose the PIG’N’PI algorithm to learn particle interactions that are consistent with the underlying physical laws. The main novelty of the proposed algorithm is in the design of the physics operator in the node part. The designed physics operator on nodes guides the edge neural network to learn the pairwise force or pairwise potential energy exactly. This design also reduces the model complexity for this machine learning algorithm by reducing the number of tunable parameters.

While our method shows a similar performance on the supervised learning task of predicting accelerations compared to the other baseline models (purely data-driven GNs), it is also able to infer the particle interactions correctly that follow the underlying physical laws. However, while the baseline models are able to be applied as simulators, they fail to infer physically consistent particle interactions that satisfy Newton’s laws. Moreover, the proposed PIG’N’PI also outperforms the other baseline models in terms of generalization ability to larger systems and robustness to significant levels of noise.

The proposed methodology can generalize well to larger particle systems. However, we have to point out that the trained model cannot extrapolate the data arbitrarily far from the training distribution. In our experiments, we found that the edge neural network converges to linear functions outside the training input space. This observation matches the discussion in ref. (38), which is an inherent limitation of MLPs.

The developed methodology will help to make a step forward in developing a flexible and robust tool for the discovery of physical laws in material mechanics. Such tools will be able to support, for example, additive manufacturing with heterogeneous materials that are particularly subject to highly varying material properties, e.g., sustainable or recycled materials (39).

## Methods

### Notations and formal task description

We use a fully connected directed graph *G* = (*V, E*) to represent the interacting particle system, where nodes *V* = {*v*_1_, *v*_2_, …, *v*_|*V*|_} correspond to the particles and the directed edges *E* = {*e_ij_*: *v_i_*, *v_j_* ∈ *V, i* ≠ *j*} correspond to particle interactions. Under this notation, *v_i_* refers to the *i*-th particle, and *e_ij_* is the directed edge from *v_j_* to *v_i_*. We use $\lbrace \boldsymbol {\eta }^t_i\rbrace _{i, t}$ to denote the observation of particle states at different time steps, where $\boldsymbol {\eta }^t_i$ is a vector describing the state of particle *v_i_* at time *t*. We note that $\boldsymbol {\eta }^t_i \in \mathbb {R}^{2d+2}$ (*d* is the space dimension) includes position $\boldsymbol {r}^t_i \in \mathbb {R}^d$, velocity $\boldsymbol {\dot{r}}^t_i \in \mathbb {R}^d$, electric charge $q_i \in \mathbb {R}$, and mass $m_i \in \mathbb {R}$. The velocity $\boldsymbol {\dot{r}}^t_i$ and acceleration $\boldsymbol {\ddot{r}}_i^t$ at time *t* are computed from the position series of particle *v_i_*. We use $\mathcal {M}_{ij}$ to denote the message from *v_j_* to *v_i_* learnt by the neural network $\hat{G}_E(\cdot ; \theta _{E})$ with parameters θ_*E*_. Our goal is to infer the pairwise force $F_{ij}^t$ and the potential energy $P_{ij}^t$ on every edge *e_ij_* at each time *t* given the observation of particle trajectories. All notations are summarized in Table S1.

### PIG’N’PI details

PIG’N’PI contains an edge part to learn the pairwise interaction and a node part to aggregate the interactions to derive node accelerations (see Fig. 1). In the edge part, we use MLPs as universal approximators (40, 41) to learn the pairwise force or pairwise potential energy. We denote this edge neural network as $\hat{G}_E(\cdot ; \theta _{E})$. $\hat{G}_E(\cdot ; \theta _{E})$ takes the vectors $\boldsymbol {\eta }_i$ and $\boldsymbol {\eta }_j$ of two nodes as input. The output $\mathcal {M}_{ij}$ of $\hat{G}_E(\cdot ; \theta _{E})$ is the inferred pairwise force $\boldsymbol {\hat{F}}_{ij}$ or potential energy $\hat{P}_{ij}$ on edge *e_ij_*, depending on the operator in the node part. We design the physics operator *G_N_*( · ) to aggregate the edge messages in the node part and derive the acceleration $\boldsymbol {\hat{\ddot{r}}}^t_i$ for every particle *v_i_* at time *t*. We optimize parameters θ_*E*_ by minimizing the mean absolute error between the predicted acceleration and the true acceleration. The objective function is given by

(3) $$\begin{eqnarray*}
\arg \min _{\theta _{E}} \mathcal {L} = \frac{1}{|\mathcal {T}_{\text{train}}|} \frac{1}{|V|} \sum _{t \in \mathcal {T}_{\text{train}}} \sum _{i=1}^{|V|} l_1(\boldsymbol {\hat{\ddot{r}}}_i^t,\boldsymbol {\ddot{r}}_i^t).
\end{eqnarray*}
$$

In the following, we explain the design of the edge neural network $\hat{G}_E(\cdot ; \theta _{E})$ and the node part operator *G_N_*( · ) in two cases: inferring the pairwise force and inferring the pairwise potential energy.

#### Learning pairwise force

We use an MLP as the edge neural network $\hat{G}_E(\cdot ; \theta _{E})$ to learn the pairwise force from *v_j_* to *v_i_* on each edge *e_ij_*. The output dimension of $\hat{G}_E(\cdot ; \theta _{E})$ is the same as the spatial dimension *d*. We first concatenate $\boldsymbol {\eta }_i^t$ and $\boldsymbol {\eta }_j^t$ which is the input of $\hat{G}_E(\cdot ; \theta _{E})$. We denote the corresponding output as $\mathcal {M}_{ij}^t \in \mathbb {R}^{d}$, e.g.,

(4) $$\begin{eqnarray*}
\mathcal {M}_{ij}^t \triangleq \hat{G}_E(\text{concat}(\boldsymbol {\eta }_i^t, \boldsymbol {\eta }_j^t); \theta _{E}).
\end{eqnarray*}
$$

According to Newton’s Second law, the net acceleration of every particle is equal to the net force divided by its mass. Hence, in the node part of PIG’N’PI, we first sum up all incoming messages $\mathcal {M}_{i} = \sum _j^{j\ne i}\mathcal {M}_{ij}$ of every particle *v_i_*, and then divide it by the mass of the particle *m_i_*. The output of *G_N_*( · ) is the predicted acceleration $\boldsymbol {\hat{\ddot{r}}}_i$ on particle *v_i_*:

(5) $$\begin{eqnarray*}
\boldsymbol {\hat{\ddot{r}}}_i^t & =& G_N(\boldsymbol {\eta }_i^t, \textstyle \mathcal {M}_{i}^t) \nonumber \\
& =& G_N(\boldsymbol {\eta }_i^t, \textstyle \sum _{j}^{j \ne i} \mathcal {M}_{ij}^t) \nonumber \\
& =& \frac{\textstyle \sum _{j}^{j \ne i} \mathcal {M}_{ij}^t}{m_i}.
\end{eqnarray*}
$$

We optimize the parameters *θ_E_* in $\hat{G}_E(\cdot ; \theta _{E})$ by minimizing the objective function Eq. (3). Through this process, the node part operator *G_N_*( · ) guides the edge neural network $\hat{G}_E(\cdot ; \theta _{E})$ to predict the pairwise force exactly, e.g.,

(6) $$\begin{eqnarray*}
\boldsymbol {\hat{F}}_{ij}^t = \mathcal {M}_{ij}^t.
\end{eqnarray*}
$$

This is illustrated in Block (B) of Fig. 2.

#### Learning pairwise potential energy

For the pairwise potential energy case, the edge neural network $\hat{G}_E(\cdot ; \theta _{E})$ is designed to output the pairwise potential energy. Here, the output dimension of $\hat{G}_E(\cdot ; \theta _{E})$ is one because the potential energy is a scalar. We still first concatenate $\boldsymbol {\eta }_i^t$ and $\boldsymbol {\eta }_j^t$ as the input of $\hat{G}_E(\cdot ; \theta _{E})$ and use MLPs as $\hat{G}_E(\cdot ; \theta _{E})$. The corresponding output $\mathcal {M}_{ij}^t \in \mathbb {R}$ is denoted as

(7) $$\begin{eqnarray*}
\mathcal {M}_{ij}^t \triangleq \hat{G}_E(\text{concat}(\boldsymbol {\eta }_i^t, \boldsymbol {\eta }_j^t); \theta _{E}).
\end{eqnarray*}
$$

We know that the net force of every particle equals to the negative partial derivative of the potential energy with respect to its position. Hence, in the node part, we first sum up all incoming messages $\mathcal {M}_{i} = \sum _j^{j\ne i}\mathcal {M}_{ij}$ for every particle *i*, then compute the negative derivative with respect to the input position and finally divide it by the mass. The final output corresponds then to the predicted acceleration. The node part operator *G_N_*( · ) for the potential energy case is given by

(8) $$\begin{eqnarray*}
\boldsymbol {\hat{\ddot{r}}}_i^t & =& G_N(\boldsymbol {\eta }_i^t, \textstyle \mathcal {M}_{i}^t) \nonumber \\
& = & G_N(\boldsymbol {\eta }_i^t, \textstyle \sum _{j}^{j\ne i} \mathcal {M}_{ij}^t) \nonumber \\
& =& - \frac{ \partial (\sum _{j}^{j \ne i} \mathcal {M}_{ij}^t)/ \partial \boldsymbol {r}_i^t }{m_i}.
\end{eqnarray*}
$$

Analogously to the force-based case, we optimize for the parameters *θ_E_* in $\hat{G}_E(\cdot ; \theta _{E})$ by minimizing the acceleration loss (Eq. 3). The node part operator *G_N_*( · ) here guides the edge neural network $\hat{G}_E(\cdot ; \theta _{E})$ to learn the pairwise potential energy exactly. The learnt message on each edge corresponds to the predicted pairwise potential energy, and the negative partial derivative is the predicted pairwise force, e.g.,

(9) $$\begin{eqnarray*}
\hat{P}_{ij} &=& \mathcal {M}_{ij} \nonumber \\
\boldsymbol {\hat{F}}_{ij} &=& - \partial \hat{P}_{ij} / \partial \boldsymbol {r}_{i} \nonumber \\
&=& - \partial \mathcal {M}_{ij} / \partial \boldsymbol {r}_{i}.
\end{eqnarray*}
$$

This is illustrated in Block (C) of Fig. 2.

We note that the commonly used ReLU activation function is not suitable as activation function in $\hat{G}_E(\cdot ; \theta _{E})$ for learning the potential energy. The reason is that we compute the partial derivative of $\mathcal {M}_{ij} = \hat{G}_E(\text{concat}(\boldsymbol {\eta }_i, \boldsymbol {\eta }_j); \theta _{E})$ to derive the predicted accelerations for every particle. The derivative should be continuous and even smooth considering physical forces. However, ReLU approximates the underlying function by piece-wise linear hyper-planes with sharp boundaries. The first-order derivative is, thus, piece-wise constant that does not change with input (21). Details on selecting the activation function in $\hat{G}_E(\cdot ; \theta _{E})$ are explained in the “Details about simulation and experiments” section.

### Details on imposing a symmetry regularization on the baseline model

As mentioned in the “Comparison of PIG’N’PI to alternative hyperparameter choices and an alternative regularized architecture” section, to ensure that the model satisfies the action–reaction physical consistency requirement, we also test an extension of the baseline model by imposing a symmetry regularization on the corresponding pairwise messages in the baseline model. This can be considered as an alternative way of imposing physical consistency. In details, let $\mathcal {M}_{ij}$ be the message from *v_j_* to *v_i_* which is the output of the edge neural network of the baseline model. In our experimental setup, the message $\mathcal {M}_{ij}$ corresponds to the force from *v_j_* to *v_i_*. We impose the symmetry regularization by adding a regularization term on the learnt messages in the objective function [Eq. (3)]. This results in the following objective function:

(10) $$\begin{eqnarray*}
\arg \min _{\theta _{E}} \mathcal {L} &=& \frac{1}{|\mathcal {T}_{\text{train}}|} \sum _{t \in \mathcal {T}_{\text{train}}} (\underbrace{\frac{1}{|V|}\sum _{i=1}^{|V|} \vert \boldsymbol {\hat{\ddot{r}}}_i^t - \boldsymbol {\ddot{r}}_i^t \vert }_{\text{Acceleration loss on nodes}}\nonumber \\
&& + \ \underbrace{\alpha \frac{1}{|E|}\sum _{i,j}^{i\ne j}\vert \mathcal {M}_{ij}^t + \mathcal {M}_{ji}^t \vert }_{\text{Symmetry regularization loss on edges}} ),
\end{eqnarray*}
$$

where *α* is a weight parameter. The original baseline model can be considered as the special case with *α* = 0 in Eq. (10). In our experiments, we evaluate the impact of the regularization term with different weights (*α* = 0.1, 1.0, 10, 100). The results are reported in Table S10.

### Details of the method to learn pairwise force introduced by ref. (34)

Reference (34) proposed a method that has a similar goal to the proposed PIG’N’PI applied to pairwise force prediction. The authors also impose Newton’s second law in the standard GN block by dividing the aggregated messages by the node property. We denote this method as GN+. The main difference between GN+ and PIG’N’PI is that GN+ treats the node property as a learnable parameter. It assigns an individual learnable scalar *w_i_* for each particle *v_i_* and predicts the acceleration of *v_i_* by dividing the aggregated incoming messages by $10^{w_i}$. The learnable scalars on all nodes representing the pairwise force are learnt together with all other parameters. It is important to point out that GN+ was designed solely for learning the pairwise force while PIG’N’PI can be applied both: to infer the pairwise forces and also the pairwise potential energy. The detailed results of GN+ are reported in Tables S2, S4, and S6, and Figs. 3D, 4, and  8.

### Details about simulation and experiments

Here, we summarize the different force functions used in our simulation. Please note that in this work, we used the same case studies as in previous work (22). However, we adapted the parameters of the particle systems slightly to make the learning more challenging.

- **Spring force**: We denote the spring constant as *k* and balance length as *L*. The pairwise force from *v_i_* to *v_j_* is $k(r_{ij} - L)\boldsymbol {n}_{ij}$ and its potential energy is 0.5*k*(*r_ij_* − *L*)^2^, where $r_{ij} = \left\Vert \boldsymbol {r}_j - \boldsymbol {r}_i\right\Vert$ is the Euclidean distance and $\boldsymbol {n}_{ij}= \frac{\boldsymbol {r}_j - \boldsymbol {r}_i}{\left\Vert \boldsymbol {r}_j - \boldsymbol {r}_i\right\Vert }$ is the unit vector pointing from *v_i_* to *v_j_*. We set *k* = 2.0 and *L* = 1.0 in our simulations.
- **Charge force:** The electric charge force from *v_i_* to *v_j_* is $-c q_i q_j \boldsymbol {n}_{ij} / r_{ij}^2$ and the potential energy is *cq_i_q_j_*/*r_ij_*, where *c* is the charge constant, and *q_i_*, *q_j_* are the electric charges. We set *c* = 1.0 in the simulation. Furthermore, to prevent any zeros in the denominator, we add a small number δ (δ = 0.01) when computing distances.
- **Orbital force:** The orbital force from *v_i_* to *v_j_* equals to $m_i m_j\boldsymbol {n}_{ij}/r_{ij}$ and the potential energy is *m_i_m_j_*ln(*r_ij_*), where *m_i_*, *m_j_* are the masses of *v_i_* and *v_j_*. We again add a small number δ (δ = 0.01) when computing distances to prevent zeros in the denominator and logarithm.
- **Discontinuous force:** We set threshold constant Θ = 2.0 such that the pairwise force is $\boldsymbol {0}$ if the Euclidean distance *r_ij_* is strictly smaller than this threshold and $(r_{ij} - 1)\boldsymbol {n}_{ij}$ otherwise. The corresponding potential is 0 if *r_ij_* is strictly smaller than this threshold and 0.5(*r_ij_* − 1)^2^ otherwise.

We intentionally omit units for variables because the simulation data can be at arbitrary scale. Moreover, the presented cases serve as proof of concept to learn the input–output relation. Further, we note that *m_i_* is sampled from the log-uniform distribution within the range [−1] ($\text{ln}(m_i) \sim \mathcal {U}(-1, 1)$). *q_i_* is uniformly sampled from the range [−1, 1]. Initial location and velocity of particles are both sampled from the normal Gaussian distribution $\mathcal {N}(0, 1)$. Each simulation contains eight particles. Each particle is associated with the corresponding features including position, velocity, mass, and charge. The target for prediction is node accelerations. Every simulation contains 10,000 time steps with step size 0.01. We randomly split the simulation steps into training dataset, validation dataset, and testing dataset with the ratio 7:1.5:1.5. We use $\mathcal {T}_{\text{train}}$, $\mathcal {T}_{\text{valid}}$, and $\mathcal {T}_{\text{test}}$ to indicate the simulation time steps corresponding to training split, validation split, and testing split. We train the model on the training dataset [optimizing the parameters *θ_E_* in $\hat{G}_E( \cdot ; \theta _{E} )$] by optimizing Eq. (3), fine-tune hyperparameters, and select the best trained model on the validation dataset and report the performance of the selected trained model on the testing dataset. For generalization tests, we re-run each simulation on 12 particles with 1500 time steps (same size as original testing dataset). The previously selected trained model with eight particles is tested on the new testing dataset.

We only fine-tune hyperparameters on the spring validation dataset and use the same hyperparameters in all experiments. We set the learning rate to 0.001, the number of hidden layers in the edge neural network to 4, the units of hidden layers to 300, max training epochs to 200. The dimension of the output layer in the edge neural network is *d* to learn the force or one to learn the potential energy. We use the Adam optimizer with the mini-batch size of 32 for the force case study and eight for the potential case study to train the model. The SiLU activation function is used in all PIG’N’PI evaluations.

## Supplementary Material

pgac264_Supplemental_File

## Data Availability

The implementation of PIG’N’PI is based on PyTorch (42) and pytorch-geometric (43) libraries. The source code is available on Gitlab: https://gitlab.ethz.ch/cmbm-public/pignpi. The data used in the experiments are generated by the numerical simulator. All data used for the experiments are included in the associated Gitlab repository: https://gitlab.ethz.ch/cmbm-public/pignpi/-/tree/main/simulation.
