# Supplementary material for: Learning physics-consistent particle interactions
Source: PNAS Nexus. 2022 Nov 18;1(5):pgac264. doi: 10.1093/pnasnexus/pgac264 (PMC9802333; doi:10.1093/pnasnexus/pgac264)
Supplement: pgac264_Supplemental_File [file pgac264_supplemental_file.pdf]

Supporting Information for

Learning Physics-Consistent Particle Interactions

Zhichao Han, David S. Kammer and Olga Fink

Corresponding Author: Olga Fink.
E-mail: olga.fink@epfl.ch

This PDF file includes:

Supporting text
Figs. S1 to S2
Tables S1 to S13
SI References

Supporting Information Text

A. Symbol table. The variable notations used in the paper are summarized in Table S1.

Table S1. Symbol notations and their meanings

notation	meaning
$G = (V, E)$	graph representation of the interacting particle system
$V = \{v_1, v_2, \ldots, v_{\lvert V\rvert}\}$	set of nodes corresponding to particles
$E = \{e_{ij} : v_i, v_j \in V, i \neq j\}$	set of edges corresponding to interactions between particles
$v_i \in V$	i-th particle
$e_{ij} \in E$	directed edge from particle v_j to particle v_i
d	spatial dimension (2 or 3)
$\boldsymbol{r}_i^t \in \mathbb{R}^d$	position of v_i at time t
$\boldsymbol{n}_{ij} \in \mathbb{R}^d$	unit vector pointing from v_i to v_j, $\boldsymbol{n}_{ij} = \frac{\boldsymbol{r}_j - \boldsymbol{r}_i}{\lVert \boldsymbol{r}_j - \boldsymbol{r}_i \rVert}$
$\dot{\boldsymbol{r}}_i^t \in \mathbb{R}^d$	velocity of v_i at time t
$q_i \in \mathbb{R}$	electric charge of particle v_i, it is a constant
$m_i \in \mathbb{R}$	mass of particle v_i, it is a constant
$\boldsymbol{\eta}_i^t \in \mathbb{R}^{2d+2}$	feature vector of particle v_i at time t, $\boldsymbol{\eta}_i^t = [\boldsymbol{r}_i^t, \dot{\boldsymbol{r}}_i^t, q_i, m_i]$
$\ddot{\boldsymbol{r}}_i^t \in \mathbb{R}^d$	true acceleration of particle v_i at time t
$\hat{\ddot{\boldsymbol{r}}}_i^t \in \mathbb{R}^d$	predicted acceleration of particle v_i at time t
$\boldsymbol{F}_{ij}^t \in \mathbb{R}^d$	true force from v_j to v_i at time t
$\hat{\boldsymbol{F}}_{ij}^t \in \mathbb{R}^d$	predicted force from v_j to v_i at time t
$P_{ij}^t \in \mathbb{R}$	true potential energy incurred by v_j on v_i at time t
$\hat{P}_{ij}^t \in \mathbb{R}$	predicted potential energy incurred by v_j on v_i at time t
$\hat{G}_E(\cdot; \theta_E)$	edge part neural network of PIG'N'PI with learnable parameters θ_E
$G_N(\cdot)$	proposed deterministic node part operator of PIG'N'PI
θ_E	learnable parameters in the edge neural network $\hat{G}_E(\cdot; \theta_E)$
$\mathcal{M}_{ij}$	learnt message from v_j to v_i output by edge neural network $\hat{G}_E(\cdot; \theta_E)$, $\mathcal{M}_{ij} \in \mathbb{R}^d$ in learning force and $\mathcal{M}_{ij} \in \mathbb{R}$ in learning potential
$\mathcal{M}_i$	sum of all incoming message on particle v_i, $\mathcal{M}_i = \sum_i^{j \neq i} \mathcal{M}_{ij}$
$\mathcal{T}_{\text{train}}$	set of time steps corresponding to the training split of simulation data
$\mathcal{T}_{\text{valid}}$	set of time steps corresponding to the validation split of simulation data
$\mathcal{T}_{\text{test}}$	set of time steps corresponding to the testing split of simulation data
$l_1(x, y)$	sum of absolute differences between each element in x and y, $l_1(x, y) = \sum_i \lvert x_i - y_i \rvert$, if x and y are vectors; or the absolute difference, $l_1(x, y) = \lvert x - y \rvert$, if if x and y are scalars
k	stiffness constant in spring simulation, we set $k = 2$
L	balance length constant in spring simulation, we set $L = 1$
c	constant in charge simulation, we set $c = 1$
Θ	threshold constant in discontinuous dataset simulation, we set $\Theta = 2$

B. Performance evaluation of learning physics-consistent particle interactions (force and potential energy). Two different performance characteristics are evaluated. First, the learning performance is evaluated and, second, the ability of the algorithms to learn the particle interactions that are consistent with the underlying physical laws.

We compute the following metrics for evaluating the performance of PIG'N'PI and the baseline model to learn the pairwise force:

$$\mathsf{MAE}_{\mathsf{acc}} = \mathsf{MAE}^{\mathrm{part}}(\hat{\ddot{\boldsymbol{r}}}, \ddot{\boldsymbol{r}}) = \frac{1}{|\mathcal{T}_{\mathrm{test}}|}\frac{1}{|V|}\sum_{t\in\mathcal{T}_{\mathrm{test}}}\sum_{i\in V} l_1(\hat{\ddot{\boldsymbol{r}}}_i^t, \ddot{\boldsymbol{r}}_i^t) \tag{1}$$

$$\mathsf{MAE}_{\mathsf{ef}} = \mathsf{MAE}^{\mathrm{inter}}(\hat{\boldsymbol{F}}, \boldsymbol{F}) = \frac{1}{|\mathcal{T}_{\mathrm{test}}|}\frac{1}{|E|}\sum_{t\in\mathcal{T}_{\mathrm{test}}}\sum_{i,j\in V}^{i\neq j} l_1(\hat{\boldsymbol{F}}_{ij}^t, \boldsymbol{F}_{ij}^t) \tag{2}$$

$$\mathsf{MAE}_{\mathsf{nf}} = \mathsf{MAE}^{\mathrm{part}}(\hat{\boldsymbol{F}}, \boldsymbol{F}) = \frac{1}{|\mathcal{T}_{\mathrm{test}}|}\frac{1}{|V|}\sum_{t\in\mathcal{T}_{\mathrm{test}}}\sum_{i\in V} l_1(\hat{\boldsymbol{F}}_i^t, \boldsymbol{F}_i^t), \text{ where } \hat{\boldsymbol{F}}_i^t = \sum_{j}^{j\neq i}\hat{\boldsymbol{F}}_{ij}^t \tag{3}$$

$$\mathsf{MAE}_{\mathsf{symm}}^{F} = \frac{1}{|\mathcal{T}_{\mathrm{test}}|}\frac{1}{|E|}\sum_{t\in\mathcal{T}_{\mathrm{test}}}\sum_{i,j\in V}^{i\neq j} l_1(\hat{\boldsymbol{F}}_{ij}^t, -\hat{\boldsymbol{F}}_{ji}^t) \tag{4}$$

where $\ddot{\boldsymbol{r}}$ and $\boldsymbol{F}$ are the ground-truth acceleration and force, and $\hat{\ddot{\boldsymbol{r}}}$ and $\hat{\boldsymbol{F}}$ are the predicted acceleration and force. Table S2 reports the performance of the baseline model and PIG'N'PI to the learn pairwise force in terms of metrics listed above (learning performance and the ability of the algorithms to learn physics-consistent particle interactions).

Table S2. Performance of PIG'N'PI and the baseline model on pairwise force prediction. Baseline$_{\mathsf{SiLU}}$ denotes the baseline with the SiLU activation function. GN+ is the method to learn pairwise force introduced by (1). Results averaged across five experiments.

		Spring dim=2	Spring dim=3	Charge dim=2	Charge dim=3	Orbital dim=2	Orbital dim=3	Discnt dim=2	Discnt dim=3
$\mathsf{MAE}_{\mathsf{acc}}$	Baseline	0.0565 ±0.0023	0.1076 ±0.0012	0.2521 ±0.0173	0.3824 ±0.0559	0.0437 ±0.0026	0.0439 ±0.0014	0.0592 ±0.0015	0.1171 ±0.0010
	Baseline$_{\mathrm{SiLU}}$	0.0258 ±0.0011	0.0476 ±0.0025	1.0326 ±1.3788	0.2092 ±0.0060	0.0187 ±0.0005	0.0196 ±0.0002	0.0249 ±0.0002	0.0508 ±0.0010
	GN+	0.0246 ±0.0047	0.0542 ±0.0047	0.1216 ±0.0099	0.1890 ±0.0111	0.0255 ±0.0023	0.0581 ±0.0004	0.0667 ±0.0174	0.2280 ±0.0951
	PIG'N'PI	**0.0206 ±0.0009**	**0.0278 ±0.0021**	**0.0425 ±0.0053**	**0.1191 ±0.0027**	**0.0202 ±0.0003**	**0.0182 ±0.0003**	**0.0227 ±0.0019**	**0.0399 ±0.0011**
$\mathsf{MAE}_{\mathsf{ef}}$	Baseline	2.3979 ±0.2095	3.8952 ±0.7178	1.1832 ±0.0955	0.6447 ±0.1118	4.1010 ±0.1467	3.5379 ±0.7571	1.6536 ±0.0640	2.5803 ±0.2886
	Baseline$_{\mathrm{SiLU}}$	4.2027 ±1.1242	5.5185 ±1.1452	2.1581 ±0.9572	1.3842 ±0.1411	3.1097 ±0.7148	1.9863 ±0.1434	2.6576 ±0.4146	4.4222 ±0.6116
	GN+	0.5724 ±0.2321	0.3638 ±0.3133	1.0248 ±0.0182	0.3137 ±0.0051	0.9372 ±0.0294	0.6943 ±0.0661	0.3714 ±0.2711	0.6974 ±0.4143
	PIG'N'PI	**0.0063 ±0.0002**	**0.0101 ±0.0007**	**0.0136 ±0.0023**	**0.0363 ±0.0015**	**0.0093 ±0.0002**	**0.0095 ±0.0001**	**0.0040 ±0.0004**	**0.0079 ±0.0002**
$\mathsf{MAE}_{\mathsf{nf}}$	Baseline	11.652 ±0.9890	20.967 ±3.8552	6.8310 ±0.5548	3.8038 ±0.7523	18.194 ±0.6884	16.677 ±3.5212	10.786 ±0.3764	15.651 ±1.7983
	Baseline$_{\mathrm{SiLU}}$	20.685 ±5.0491	29.824 ±6.2007	12.480 ±5.4145	8.7533 ±0.9595	13.644 ±3.1699	9.2546 ±0.6675	17.430 ±2.7127	27.149 ±3.8191
	GN+	2.7639 ±1.1198	1.9370 ±1.6941	5.9332 ±0.1038	1.6546 ±0.0299	3.9950 ±0.1166	3.1841 ±0.2858	2.4280 ±1.8009	4.2270 ±2.6246
	PIG'N'PI	**0.0219 ±0.0010**	**0.0292 ±0.0022**	**0.0488 ±0.0059**	**0.1317 ±0.0033**	**0.0260 ±0.0005**	**0.0233 ±0.0004**	**0.0239 ±0.0020**	**0.0419 ±0.0011**
$\mathsf{MAE}_{\mathsf{symm}}^{F}$	Baseline	1.1099 ±0.0785	1.7452 ±0.0467	0.1248 ±0.0137	0.6938 ±0.2670	2.2074 ±0.1852	1.7684 ±0.0941	0.9399 ±0.0257	1.4118 ±0.0722
	Baseline$_{\mathrm{SiLU}}$	2.1473 ±0.1366	3.1809 ±0.5156	2.1585 ±1.8080	2.1378 ±0.2803	0.8103 ±0.1062	0.8877 ±0.0584	1.6121 ±0.2357	2.4231 ±0.3908
	GN+	0.0400 ±0.0437	0.0756 ±0.0284	0.1013 ±0.0082	0.0315 ±0.0033	1.0831 ±0.0733	0.8068 ±0.1251	0.0102 ±0.0041	0.0975 ±0.0927
	PIG'N'PI	**0.0075 ±0.0003**	**0.0133 ±0.0008**	**0.0185 ±0.0036**	**0.0345 ±0.0017**	**0.0136 ±0.0004**	**0.0134 ±0.0004**	**0.0026 ±0.0004**	**0.0066 ±0.0001**

We use the following metrics for evaluating the performance to learn pairwise potential energy:

$$\mathsf{MAE}_{\mathsf{acc}} = \mathsf{MAE}^{\text{part}}(\hat{\ddot{\boldsymbol{r}}}, \ddot{\boldsymbol{r}}) = \frac{1}{|\mathcal{T}_{\text{test}}|}\frac{1}{|V|}\sum_{t\in\mathcal{T}_{\text{test}}}\sum_{i\in V} l_1(\hat{\ddot{\boldsymbol{r}}}_i^t, \ddot{\boldsymbol{r}}_i^t) \qquad [5]$$

$$\mathsf{MAE}_{\Delta\mathsf{ep}} = \mathsf{MAE}^{\text{inter}}(\hat{P} - \hat{P}^0, P - P^0) = \frac{1}{|\mathcal{T}_{\text{test}}|}\frac{1}{|E|}\sum_{t\in\mathcal{T}_{\text{test}}}\sum_{i,j\in V}^{i\neq j} l_1(\hat{P}_{ij}^t - \hat{P}_{ij}^0, P_{ij}^t - P_{ij}^0) \qquad [6]$$

$$\mathsf{MAE}_{\Delta\mathsf{np}} = \mathsf{MAE}^{\text{part}}(\hat{P} - \hat{P}^0, P - P^0) = \frac{1}{|\mathcal{T}_{\text{test}}|}\frac{1}{|V|}\sum_{t\in\mathcal{T}_{\text{test}}}\sum_{i\in|V|} l_1(\sum_{j}^{j\neq i}\hat{P}_{ij}^t - \sum_{j}^{j\neq i}\hat{P}_{ij}^0, P_i^t - P_i^0) \qquad [7]$$

$$\mathsf{MAE}_{\mathsf{ef}} = \mathsf{MAE}^{\text{inter}}(\hat{\boldsymbol{F}}, \boldsymbol{F}) = \frac{1}{|\mathcal{T}_{\text{test}}|}\frac{1}{|E|}\sum_{t\in\mathcal{T}_{\text{test}}}\sum_{i,j\in V}^{i\neq j} l_1(\hat{\boldsymbol{F}}_{ij}^t, \boldsymbol{F}_{ij}^t), \text{ where } \hat{\boldsymbol{F}}_{ij}^t = -\frac{\partial \hat{P}_{ij}^t}{\partial \boldsymbol{r}_i^t} \qquad [8]$$

$$\mathsf{MAE}_{\mathsf{nf}} = \mathsf{MAE}^{\text{part}}(\hat{\boldsymbol{F}}, \boldsymbol{F}) = \frac{1}{|\mathcal{T}_{\text{test}}|}\frac{1}{|V|}\sum_{t\in\mathcal{T}_{\text{test}}}\sum_{i\in V} l_1(\hat{\boldsymbol{F}}_i^t, \boldsymbol{F}_i^t), \text{ where } \hat{\boldsymbol{F}}_i^t = -\frac{\partial \sum_j^{j\neq i}\hat{P}_{ij}^t}{\partial \boldsymbol{r}_i^t} \qquad [9]$$

$$\mathsf{MAE}_{\mathsf{symm}}^P = \frac{1}{|\mathcal{T}_{\text{test}}|}\frac{1}{|E|}\sum_{t\in\mathcal{T}_{\text{test}}}\sum_{i,j\in V}^{i\neq j} l_1(\hat{P}_{ij}^t, \hat{P}_{ji}^t) \qquad [10]$$

where $\ddot{\boldsymbol{r}}$, $\boldsymbol{F}$ and P are the ground-truth accelerations, forces and potentials, $\hat{\ddot{\boldsymbol{r}}}$, $\hat{\boldsymbol{F}}$ and $\hat{P}$ are the predictions computed from Eq. (8)-(9). Table S3 reports the performance of baseline model and PIG'N'PI to learn pairwise potential energy.

Table S3. Performance evaluation of PIG'N'PI and the baseline model on the pairwise potential energy learning task. Baseline$_{\text{SiLU}}$ denotes the baseline model with SiLU activation function. We report the error of predicting the potential energy and its first-order derivative which corresponds to the inter-particle force. Results averaged across five experiments.

		Spring dim=2	Spring dim=3	Charge dim=2	Charge dim=3	Orbital dim=2	Orbital dim=3	Discnt dim=2	Discnt dim=3
MAE_{acc}	Baseline	1.4841 ±0.0064	1.9996 ±0.0253	2.9127 ±0.0844	0.5959 ±0.0087	2.4585 ±0.0399	1.0113 ±0.0100	0.4532 ±0.0186	0.7222 ±0.0168
	Baseline$_{\text{SiLU}}$	1.4094 ±0.0842	1.8721 ±0.0567	4.5466 ±0.1088	0.6047 ±0.0169	2.2880 ±0.0231	0.9044 ±0.0125	0.3923 ±0.0058	0.6554 ±0.0132
	PIG'N'PI	**0.0076 ±0.0003**	**0.0099 ±0.0007**	**0.0225 ±0.0012**	**0.1088 ±0.0079**	**0.0090 ±0.0004**	**0.0091 ±0.0004**	**0.0089 ±0.0002**	**0.0150 ±0.0022**
MAE_{ef}	Baseline	1.7644 ±0.0104	2.4864 ±0.0104	1.5492 ±0.0553	0.5105 ±0.1459	3.0739 ±0.0580	1.9313 ±0.0076	0.7243 ±0.0111	1.1630 ±0.0063
	Baseline$_{\text{SiLU}}$	2.2647 ±0.0420	2.7155 ±0.0333	2.2720 ±0.2375	1.0911 ±0.1684	3.4747 ±0.0963	2.1853 ±0.0321	1.1977 ±0.0359	1.6008 ±0.0311
	PIG'N'PI	**0.0023 ±0.0001**	**0.0037 ±0.0003**	**0.0080 ±0.0006**	**0.0223 ±0.0013**	**0.0058 ±0.0011**	**0.0053 ±0.0005**	**0.0016 ±3.4E-5**	**0.0030 ±0.0004**
MAE_{nf}	Baseline	8.3353 ±0.0627	13.1721 ±0.0716	9.3034 ±0.3807	2.6295 ±0.6315	14.1222 ±0.3045	9.1354 ±0.0546	4.6927 ±0.0584	6.7873 ±0.0566
	Baseline$_{\text{SiLU}}$	10.3496 ±0.1832	14.1069 ±0.1588	10.6447 ±1.0175	5.1109 ±0.6467	16.2250 ±0.5242	10.5310 ±0.1533	7.9723 ±0.2558	9.6495 ±0.1291
	PIG'N'PI	**0.0080 ±0.0003**	**0.0104 ±0.0007**	**0.0261 ±0.0014**	**0.1212 ±0.0085**	**0.0115 ±0.0006**	**0.0118 ±0.0005**	**0.0098 ±0.0002**	**0.0160 ±0.0023**
$\text{MAE}_{\triangle\text{ep}}$	Baseline	0.9588 ±0.0048	1.1007 ±0.0058	0.4656 ±0.0046	0.3734 ±0.2349	1.3174 ±0.0442	1.1298 ±0.0088	0.5979 ±0.0210	0.8568 ±0.0167
	Baseline$_{\text{SiLU}}$	1.2192 ±0.0296	1.2418 ±0.0226	1.6590 ±0.3484	1.3053 ±0.2559	1.4173 ±0.0648	1.1852 ±0.0376	0.9872 ±0.0395	1.0427 ±0.0415
	PIG'N'PI	**0.0005 ±1.4E-5**	**0.0016 ±0.0003**	**0.0096 ±0.0009**	**0.0156 ±0.0015**	**0.0048 ±0.0017**	**0.0031 ±0.0006**	**0.2197 ±0.0001**	**0.2344 ±0.0001**
$\text{MAE}_{\triangle\text{np}}$	Baseline	4.0389 ±0.2410	5.5378 ±0.1083	1.5875 ±0.0363	1.6498 ±1.2898	4.9960 ±0.3848	4.7247 ±0.0448	2.9314 ±0.2362	3.7998 ±0.2532
	Baseline$_{\text{SiLU}}$	5.3748 ±0.6218	6.0889 ±0.2634	6.3113 ±1.7498	6.2894 ±0.8594	5.4304 ±0.5369	5.1887 ±0.1327	5.2514 ±0.3821	4.9127 ±0.5259
	PIG'N'PI	**0.0016 ±0.0001**	**0.0062 ±0.0012**	**0.0179 ±0.0011**	**0.0381 ±0.0037**	**0.0129 ±0.0046**	**0.0074 ±0.0009**	**0.9319 ±0.0005**	**0.9322 ±0.0004**
$\text{MAE}^{P}_{\text{symm}}$	Baseline	0.5641 ±0.0119	0.4931 ±0.0056	0.1094 ±0.0055	0.2663 ±0.3068	0.8017 ±0.0528	0.4261 ±0.0121	0.3538 ±0.0222	0.3474 ±0.0106
	Baseline$_{\text{SiLU}}$	1.1668 ±0.0281	0.9798 ±0.0735	1.8888 ±0.6032	1.2961 ±0.3374	1.0689 ±0.0443	0.7108 ±0.0394	0.8908 ±0.0287	0.8612 ±0.0305
	PIG'N'PI	**0.0007 ±0.0001**	**0.0025 ±0.0008**	**0.0074 ±0.0010**	**0.0062 ±0.0005**	**0.0252 ±0.0065**	**0.0422 ±0.0144**	**0.0003 ±2.1E-5**	**0.0005 ±2.6E-5**

C. Evaluation of the generalization ability on learning the pairwise force and potential energy. We evaluate the generalization ability of the baseline model, GN+ and PIG'N'PI by first training the models on an eight-particle system and then evaluating their performance on a 12-particle system. We evaluate the performance of baseline model, GN+ and PIG'N'PI on the pairwise force learning task (Table S4) and baseline model and PIG'N'PI on the pairwise potential energy learning task (Table S5) because GN+ is only designed for learning the pairwise force. Furthermore, a limitation of GN+ is, after training, it cannot be generalized to predict the acceleration for a new system. The reason is the learnt node property is specifically associated to the system used for training. We need to train GN+ from scratch again to predict the acceleration for a new system.

Table S4. Evaluation of the generalization ability on the pairwise force learning task. Models are trained on a eight-particle system and then tested on a 12-particle system. Results averaged across five experiments. Note that GN+ cannot be generalized to predict the acceleration because of the learnt node property.

		Spring dim=2	Spring dim=3	Charge dim=2	Charge dim=3	Orbital dim=2	Orbital dim=3	Discnt dim=2	Discnt dim=3
MAE_{acc}	Baseline	0.2790 ±0.0402	0.5664 ±0.0630	1.0363 ±0.0780	2.2038 ±0.3393	0.1007 ±0.0096	0.1497 ±0.0166	0.2067 ±0.0217	0.3705 ±0.0274
	GN+	- -	- -	- -	- -	- -	- -	- -	- -
	PIG'N'PI	**0.0449 ±0.0014**	**0.0680 ±0.0034**	**0.3561 ±0.0481**	**0.5467 ±0.0441**	**0.0413 ±0.0020**	**0.0407 ±0.0010**	**0.0489 ±0.0042**	**0.0726 ±0.0014**
MAE_{ef}	Baseline	2.1514 ±0.1950	4.2343 ±0.7791	0.6920 ±0.0616	0.6283 ±0.1043	3.3921 ±0.1321	3.3837 ±0.7063	1.6555 ±0.1018	2.7026 ±0.3773
	GN+	0.5563 ±0.2231	0.3990 ±0.3266	0.5907 ±0.0102	0.3177 ±0.0053	0.6381 ±0.0030	0.5792 ±0.0328	0.3717 ±0.2698	0.7442 ±0.5024
	PIG'N'PI	**0.0087 ±0.0002**	**0.0149 ±0.0008**	**0.0481 ±0.0065**	**0.0697 ±0.0043**	**0.0111 ±0.0008**	**0.0113 ±0.0004**	**0.0052 ±0.0005**	**0.0092 ±0.0002**
MAE_{nf}	Baseline	14.7789 ±1.4639	34.1458 ±6.3003	5.8505 ±0.5123	5.2545 ±1.0215	21.1451 ±0.9028	23.0175 ±4.7677	16.5313 ±1.0373	25.0296 ±3.6511
	GN+	3.8516 ±1.5444	3.2087 ±2.6770	4.9887 ±0.0858	2.3699 ±0.0388	4.0284 ±0.0148	3.8214 ±0.2360	3.7130 ±2.7434	6.5094 ±4.1421
	PIG'N'PI	**0.0443 ±0.0012**	**0.0665 ±0.0033**	**0.4178 ±0.0614**	**0.5564 ±0.0425**	**0.0476 ±0.0029**	**0.0451 ±0.0011**	**0.0477 ±0.0039**	**0.0730 ±0.0016**
$\text{MAE}^{F}_{\text{symm}}$	Baseline	1.0060 ±0.0711	1.6034 ±0.0494	0.1059 ±0.0158	0.6677 ±0.2549	1.6018 ±0.1370	1.6047 ±0.0858	0.8586 ±0.0239	1.2154 ±0.0622
	GN+	0.0452 ±0.0372	0.0731 ±0.0250	0.0775 ±0.0065	0.0357 ±0.0032	0.7903 ±0.0542	0.7328 ±0.1140	0.0114 ±0.0053	0.2427 ±0.3709
	PIG'N'PI	**0.0108 ±0.0003**	**0.0197 ±0.0008**	**0.0733 ±0.0125**	**0.0614 ±0.0021**	**0.0158 ±0.0013**	**0.0149 ±0.0005**	**0.0039 ±0.0006**	**0.0086 ±0.0003**

Table S5. Evaluation of the generalization ability on the potential energy learning task. Models are trained on a eight-particle system and then tested on a 12-particle system. Results averaged across five experiments. Here, the comparison model does not contain GN+ because it is only designed for learning force.

		Spring dim=2	Spring dim=3	Charge dim=2	Charge dim=3	Orbital dim=2	Orbital dim=3	Discnt dim=2	Discnt dim=3
MAE_{acc}	Baseline	6.7336 ±0.0626	14.697 ±0.3470	6.2643 ±0.7284	3.5436 ±0.3228	5.5236 ±0.0863	6.0802 ±0.0930	2.6173 ±0.1238	5.4259 ±0.3450
	PIG'N'PI	**0.0180 ±0.0018**	**0.0238 ±0.0025**	**1.1900 ±0.3611**	**0.5542 ±0.0644**	**0.0760 ±0.0167**	**0.0995 ±0.0198**	**0.0215 ±0.0017**	**0.0311 ±0.0020**
MAE_{ef}	Baseline	1.7397 ±0.0114	2.6430 ±0.0120	0.8552 ±0.0267	0.5264 ±0.1457	2.3356 ±0.0487	1.7652 ±0.0116	0.7328 ±0.0148	1.1855 ±0.0213
	PIG'N'PI	**0.0034 ±0.0002**	**0.0049 ±0.0004**	**0.1385 ±0.0357**	**0.0631 ±0.0065**	**0.0144 ±0.0020**	**0.0186 ±0.0039**	**0.0022 ±0.0001**	**0.0040 ±0.0003**
MAE_{nf}	Baseline	11.819 ±0.0599	21.083 ±0.1454	7.5812 ±0.2963	3.7500 ±0.7783	15.014 ±0.3101	12.254 ±0.1030	7.2370 ±0.1482	10.400 ±0.0918
	PIG'N'PI	**0.0173 ±0.0013**	**0.0233 ±0.0022**	**1.3505 ±0.3867**	**0.5486 ±0.0646**	**0.0704 ±0.0138**	**0.0930 ±0.0171**	**0.0204 ±0.0013**	**0.0317 ±0.0022**
$\text{MAE}_{\Delta\text{ep}}$	Baseline	2.1921 ±0.0082	3.1820 ±0.0284	0.5311 ±0.0044	0.4822 ±0.1159	0.9830 ±0.0112	0.9161 ±0.0032	0.7449 ±0.0165	1.7985 ±0.0182
	PIG'N'PI	**0.0022 ±0.0002**	**0.0033 ±0.0004**	**0.0516 ±0.0072**	**0.0428 ±0.0037**	**0.0086 ±0.0007**	**0.0106 ±0.0023**	**0.2238 ±0.0001**	**0.2415 ±0.0003**
$\text{MAE}_{\Delta\text{np}}$	Baseline	19.256 ±0.0530	28.514 ±0.4680	2.0988 ±0.0294	1.9149 ±1.0773	7.1296 ±0.2341	7.4346 ±0.1342	5.6309 ±0.2235	15.478 ±0.2467
	PIG'N'PI	**0.0137 ±0.0022**	**0.0166 ±0.0049**	**0.2937 ±0.0485**	**0.1668 ±0.0173**	**0.0415 ±0.0063**	**0.0503 ±0.0105**	**1.3273 ±0.0009**	**1.8850 ±0.0028**
$\text{MAE}^{P}_{\text{symm}}$	Baseline	0.4309 ±0.0094	0.5237 ±0.0743	0.0481 ±0.0048	0.2303 ±0.2488	0.6652 ±0.0404	0.3765 ±0.0188	0.3753 ±0.0190	0.3894 ±0.0914
	PIG'N'PI	**0.0010 ±0.0001**	**0.0033 ±0.0007**	**0.0159 ±0.0027**	**0.0124 ±0.0011**	**0.0219 ±0.0065**	**0.0336 ±0.0099**	**0.0004 ±0.0001**	**0.0009 ±0.0001**

D. Potential energy prediction in the discontinuous dataset. Here, we take a closer look at the discontinuous dataset as it presented a particularly large $\mathsf{MAE}_{\Delta\mathsf{ep}}$ for PIG'N'PI predictions compared to the other (continuous) datasets (Fig. 6). The potential energy field P presents a discontinuity at $r = 2$ (see Fig. S1(A)), where $P = 0$ for $r < 2$ and $P \geq 0.5$ for $r \geq 2$. PIG'N'PI, however, appears to infer a continuous potential function $P_{\text{PIG'N'PI}}$ (see Fig. S1(B)) that presents similar trades to the ground-truth but without the discontinuity. In fact, PIG'N'PI infers the shape of the potential energy function independently in the two areas separated by $r = 2$ without learning the absolute value of the potential energy (see $P_{\text{PIG'N'PI}} - P$ in Fig. S1(C)). The reported mean values of $P_{\text{PIG'N'PI}} - P$ for each area (see Fig. S1(C)) are relatively large indicating the error in the absolute value, whereas the values for the standard deviation are small in both areas showing that PIG'N'PI infers well the shape of the potential (*i.e.*, the derivative of the potential).

Note that the difference in the mean values between the two areas suggests that the absolute value is differently incorrect in the two areas. This explains why the $\mathsf{MAE}_{\Delta\mathsf{ep}}$ of PIG'N'PI is larger on the discontinuous dataset (Fig. 6) compared to other datasets. Here, PIG'N'PI learns the shape of the potential energy function in two ranges separately, and hence introduces a different discontinuity, which leads to an arbitrary constant that is integrated into the $\mathsf{MAE}_{\Delta\mathsf{ep}}$ computation over the *entire* space. Therefore, the increased value of $\mathsf{MAE}_{\Delta\mathsf{ep}}$ simply indicates that the discontinuity in the potential cannot be normalized-out with a measure of the relative potential energy as for the continuous datasets.

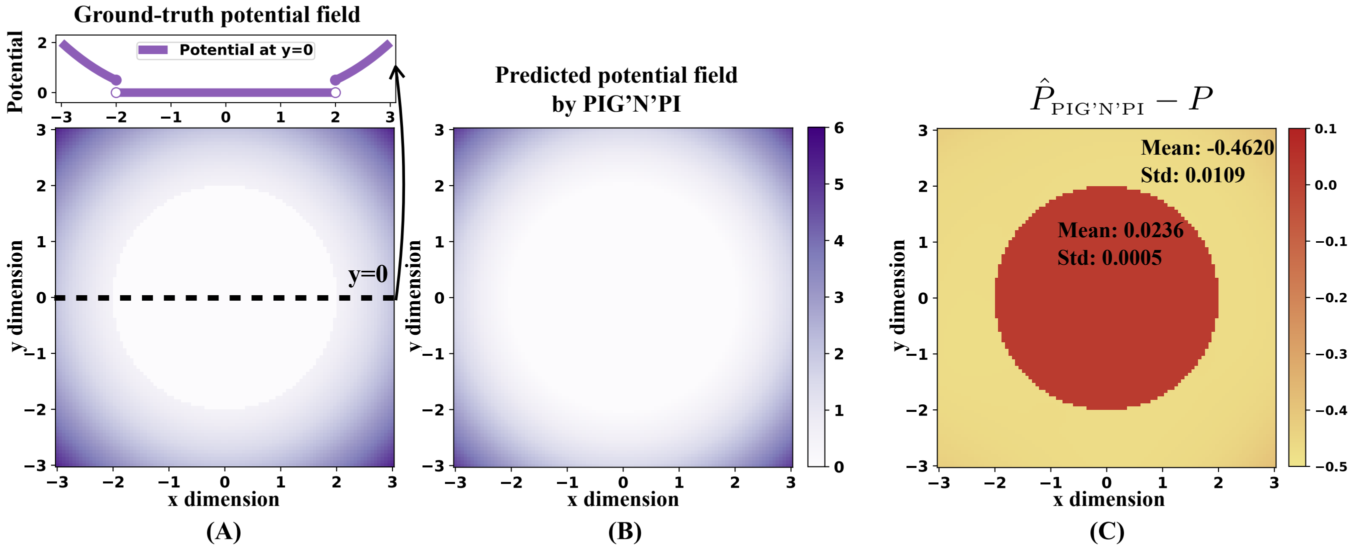

Fig. S1. Ground-truth potential energy and predicted potential energy of PIG'N'PI for the Discontinuous dataset. (A) The ground-truth discontinuous potential field around a fixed particle at center. The potential between two particles is discontinuous at distance $r = 2$. (top) Cross-section of the potential at $y = 0$. (B) The predicted potential field by PIG'N'PI. (C) Difference between the potential field predicted by PIG'N'PI and the ground-truth: $\hat{P}_{\text{PIG'N'PI}} - P$. The mean value and standard deviation are computed separately for the two areas limited by the position of the discontinuity in the potential, $r = 2$.

E. Performance evaluation for the LJ-argon dataset. Table S6 and Table S7 report the performance of PIG'N'PI to learn pairwise force and pairwise potential energy. $\mathsf{MAE}_{\mathsf{acc}}$, $\mathsf{MAE}_{\Delta\mathsf{ep}}$, $\mathsf{MAE}_{\Delta\mathsf{np}}$, $\mathsf{MAE}_{\mathsf{ef}}$, $\mathsf{MAE}_{\mathsf{nf}}$, $\mathsf{MAE}^{F}_{\mathsf{symm}}$ and $\mathsf{MAE}^{P}_{\mathsf{symm}}$ are defined same as before (see Sec. A and Sec. B). We also compute the division between each error and the average of its corresponding ground-truth as the relative error. For example, the relative $\mathsf{MAE}_{\mathsf{acc}} = \mathsf{MAE}_{\mathsf{acc}} / \frac{1}{N}\frac{1}{T}\sum_{i=1}^{N}\sum_{t=1}^{T}|\ddot{\boldsymbol{r}}_i^t|$. Note that the relative $\mathsf{MAE}_{\mathsf{acc}}$ equals to the relative $\mathsf{MAE}_{\mathsf{nf}}$ because all particles have the same mass. Baseline$_{\alpha=*}$ refers to the baseline model with symmetry regularization. See Sec. C for the details of imposing the symmetry regularization into baseline. We can find that symmetry regularization makes the baseline model perform better in terms of $\mathsf{MAE}_{\mathsf{ef}}$. However, PIG'N'PI is still significantly better than the extended baseline. Furthermore, when evaluate the models to learn pairwise force, we also test the method GN+ proposed by (1) to learn pairwise force (see Sec. D). Considering particles in this dataset have the same mass, we also test a variant of GN+ such that we assign all nodes with a unique learnable scalar. We denote this variant as GN+$_{\mathrm{uni}}$. We can see that GN+$_{\mathrm{uni}}$ is better than the baseline and GN+. However, PIG'N'PI still outperforms GN+$_{\mathrm{uni}}$ by more than one order of magnitude, especially if we look at the $\mathsf{MAE}_{\mathsf{ef}}$ which measures the quality of the predicted pariwise force.

Table S6. Evaluation of the performance to learn pairwise force for the LJ-argon dataset. Results averaged across five experiments.

	$\mathsf{MAE}_{\mathsf{acc}}$ (Å/ps^2)	Relative $\mathsf{MAE}_{\mathsf{acc}}$	$\mathsf{MAE}_{\mathsf{nf}}$ (meV/Å)	Relative $\mathsf{MAE}_{\mathsf{nf}}$	$\mathsf{MAE}_{\mathsf{ef}}$ (meV/Å)	Relative $\mathsf{MAE}_{\mathsf{ef}}$	$\mathsf{MAE}^{F}_{\mathsf{symm}}$ (meV/Å)	Relative $\mathsf{MAE}^{F}_{\mathsf{symm}}$
Baseline	0.4230 ±0.0206	2.66% ±0.13%	1.7493 ±0.0850	2.66% ±0.13%	7.2635 ±0.8811	269.41% ±32.68%	0.3885 ±0.0425	14.41% ±1.58%
Baseline$_{\alpha=0.1}$	0.6326 ±0.0381	3.97% ±0.24%	2.6160 ±0.1576	3.97% ±0.24%	3.0155 ±0.3660	111.85% ±13.57%	0.1263 ±0.0050	4.68% ±0.19%
Baseline$_{\alpha=1}$	0.8022 ±0.0706	5.04% ±0.44%	3.3171 ±0.2918	5.04% ±0.44%	2.7034 ±0.2164	100.27% ±8.02%	0.0879 ±0.0126	3.26% ±0.47%
Baseline$_{\alpha=10}$	15.1633 ±1.7219	95.24% ±10.81%	62.7044 ±7.1206	95.24% ±10.81%	2.6961 ±0.0001	100.00% ±2.94E-5	0.0069 ±0.0048	0.26% ±0.18%
Baseline$_{\alpha=100}$	15.9832 ±0.0027	100.39% ±0.02%	66.0951 ±0.0110	100.39% ±0.02%	2.6961 ±0.0000	100.00% ±0.00E0	**1.64E-8 ±1.18E-8**	**6.07E-9 ±4.37E-9**
GN+	15.7991 ±0.0001	99.23% ±3.54E-06	65.3337 ±0.0002	99.23% ±3.54E-06	7.7990 ±0.2822	289.27% ±10.47%	1.1210 ±0.0599	41.58% ±2.22%
GN+$_{\mathrm{uni}}$	0.3832 ±0.1498	2.41% ±0.94%	1.5848 ±0.6196	2.41% ±0.94%	0.5799 ±0.3035	21.51% ±11.26%	0.0155 ±0.0092	0.58% ±0.34%
PIG'N'PI	**0.0600 ±0.0020**	**0.38% ±0.01%**	**0.2483 ±0.0081**	**0.38% ±0.01%**	**0.0194 ±0.0006**	**0.72% ±0.02%**	0.0270 ±0.0008	1.00% ±0.03%

Table S7. Evaluation of the performance to learn pairwise potential energy for the LJ-argon dataset. Results averaged across five experiments.

	$\mathsf{MAE}_{\mathsf{acc}}$ (Å/ps^2)	Relative $\mathsf{MAE}_{\mathsf{acc}}$	$\mathsf{MAE}_{\mathsf{nf}}$ (meV/Å)	Relative $\mathsf{MAE}_{\mathsf{nf}}$	$\mathsf{MAE}_{\mathsf{ef}}$ (meV/Å)	Relative $\mathsf{MAE}_{\mathsf{ef}}$	$\mathsf{MAE}_{\mathsf{ep}}$ (meV)	Relative $\mathsf{MAE}_{\mathsf{ep}}$	$\mathsf{MAE}_{\mathsf{np}}$ (meV)	Relative $\mathsf{MAE}_{\mathsf{np}}$	$\mathsf{MAE}^{P}_{\mathsf{symm}}$ (meV)	Relative $\mathsf{MAE}^{P}_{\mathsf{symm}}$
Baseline	10.8064 ±0.0113	67.87% ±0.07%	44.6875 ±0.0467	67.87% ±0.07%	73.4575 ±6.3486	2725% ±236%	13.9532 ±1.1882	1051% ±89.47%	403.866 ±34.9743	510% ±44.2%	21.6873 ±1.8809	1633% ±141.6%
PIG'N'PI	**0.0714 ±0.0057**	**0.45% ±0.04%**	**0.2951 ±0.0238**	**0.45% ±0.04%**	**0.0217 ±0.0016**	**0.81% ±0.06%**	**0.0176 ±0.0014**	**1.33% ±0.11%**	**0.4428 ±0.1061**	**0.56% ±0.13%**	**0.0174 ±0.0020**	**1.31% ±0.15%**

F. Evaluation of PIG'N'PI with different activation functions to learn force. Table S8 reports the performance of PIG'N'PI with different activation functions to learn pairwise force.

Table S8. Quality of pairwise force prediction of PIG'N'PI with different activation functions. Results averaged across five experiments.

		Spring dim=2	Spring dim=3	Charge dim=2	Charge dim=3	Orbital dim=2	Orbital dim=3	Discnt dim=2	Discnt dim=3
MAE_{acc}	SiLU	0.0206 ±0.0009	0.0278 ±0.0021	0.0425 ±0.0053	0.1191 ±0.0027	0.0202 ±0.0003	0.0182 ±0.0003	0.0227 ±0.0019	0.0399 ±0.0011
	ReLU	0.0339 ±0.0007	0.0524 ±0.0009	0.1528 ±0.0039	0.2058 ±0.0066	0.0402 ±0.0028	0.0399 ±0.0003	0.0463 ±0.0020	0.0868 ±0.0026
	GELU	0.0171 ±0.0009	0.0189 ±0.0007	0.0401 ±0.0017	0.1247 ±0.0077	0.0212 ±0.0008	0.0191 ±0.0004	0.0232 ±0.0030	0.0388 ±0.0013
	tanh	0.0234 ±0.0004	0.0645 ±0.0003	0.1713 ±0.0388	0.3252 ±0.0267	0.0415 ±0.0002	0.0860 ±0.0015	0.0646 ±0.0148	0.1046 ±0.0007
	sigmoid	0.0597 ±0.0046	0.1618 ±0.0173	1.1555 ±0.0121	0.2747 ±0.0455	0.0381 ±0.0042	0.0421 ±0.0024	0.1053 ±0.0014	0.2588 ±0.0235
	softplus	0.0228 ±0.0011	0.0354 ±0.0018	0.0647 ±0.0071	0.0933 ±0.0045	0.0293 ±0.0018	0.0302 ±0.0012	0.0508 ±0.0017	0.1720 ±0.0145
	LeakyReLU	0.0326 ±0.0009	0.0545 ±0.0016	0.1477 ±0.0036	0.2212 ±0.0059	0.0387 ±0.0018	0.0396 ±0.0005	0.0494 ±0.0014	0.0910 ±0.0032
MAE_{ef}	SiLU	0.0063 ±0.0002	0.0101 ±0.0007	0.0136 ±0.0023	0.0363 ±0.0015	0.0093 ±0.0002	0.0095 ±0.0001	0.0040 ±0.0004	0.0079 ±0.0002
	ReLU	0.0146 ±0.0004	0.0247 ±0.0006	0.0379 ±0.0013	0.0574 ±0.0022	0.0179 ±0.0012	0.0201 ±0.0004	0.0088 ±0.0003	0.0202 ±0.0006
	GELU	0.0059 ±0.0003	0.0079 ±0.0003	0.0120 ±0.0005	0.0347 ±0.0020	0.0097 ±0.0004	0.0108 ±0.0003	0.0041 ±0.0006	0.0077 ±0.0002
	tanh	0.0096 ±0.0003	0.0279 ±0.0002	0.0363 ±0.0068	0.0920 ±0.0075	0.0171 ±0.0003	0.0350 ±0.0007	0.0137 ±0.0033	0.0257 ±0.0003
	sigmoid	0.0166 ±0.0014	0.0477 ±0.0040	0.2129 ±0.0020	0.0607 ±0.0079	0.0160 ±0.0018	0.0172 ±0.0011	0.0211 ±0.0003	0.0654 ±0.0075
	softplus	0.0067 ±0.0002	0.0118 ±0.0005	0.0181 ±0.0021	0.0257 ±0.0011	0.0121 ±0.0006	0.0141 ±0.0003	0.0098 ±0.0004	0.0363 ±0.0028
	LeakyReLU	0.0139 ±0.0006	0.0258 ±0.0011	0.0352 ±0.0008	0.0578 ±0.0023	0.0170 ±0.0009	0.0197 ±0.0006	0.0096 ±0.0004	0.0215 ±0.0008
MAE_{nf}	SiLU	0.0219 ±0.0010	0.0292 ±0.0022	0.0488 ±0.0059	0.1317 ±0.0033	0.0260 ±0.0005	0.0233 ±0.0004	0.0239 ±0.0020	0.0419 ±0.0011
	ReLU	0.0358 ±0.0007	0.0552 ±0.0009	0.1694 ±0.0046	0.2246 ±0.0070	0.0483 ±0.0033	0.0494 ±0.0004	0.0489 ±0.0019	0.0911 ±0.0023
	GELU	0.0182 ±0.0009	0.0202 ±0.0007	0.0460 ±0.0017	0.1366 ±0.0078	0.0270 ±0.0010	0.0249 ±0.0006	0.0244 ±0.0032	0.0402 ±0.0012
	tanh	0.0249 ±0.0006	0.0682 ±0.0003	0.1975 ±0.0444	0.3719 ±0.0303	0.0510 ±0.0003	0.1166 ±0.0018	0.0682 ±0.0157	0.1097 ±0.0009
	sigmoid	0.0629 ±0.0048	0.1673 ±0.0179	1.3848 ±0.0148	0.3233 ±0.0533	0.0496 ±0.0054	0.0553 ±0.0038	0.1111 ±0.0014	0.2722 ±0.0243
	softplus	0.0239 ±0.0012	0.0367 ±0.0018	0.0753 ±0.0086	0.1038 ±0.0051	0.0366 ±0.0016	0.0390 ±0.0013	0.0541 ±0.0018	0.1829 ±0.0161
	LeakyReLU	0.0344 ±0.0010	0.0573 ±0.0018	0.1634 ±0.0044	0.2411 ±0.0058	0.0464 ±0.0022	0.0489 ±0.0005	0.0520 ±0.0016	0.0951 ±0.0031
MAE^{F}_{symm}	SiLU	0.0075 ±0.0003	0.0133 ±0.0008	0.0185 ±0.0036	0.0345 ±0.0017	0.0136 ±0.0004	0.0134 ±0.0004	0.0026 ±0.0004	0.0066 ±0.0001
	ReLU	0.0205 ±0.0006	0.0350 ±0.0009	0.0459 ±0.0013	0.0477 ±0.0018	0.0256 ±0.0016	0.0285 ±0.0007	0.0104 ±0.0004	0.0248 ±0.0008
	GELU	0.0074 ±0.0003	0.0108 ±0.0003	0.0151 ±0.0007	0.0311 ±0.0013	0.0138 ±0.0006	0.0155 ±0.0005	0.0031 ±0.0003	0.0071 ±0.0003
	tanh	0.0128 ±0.0005	0.0367 ±0.0002	0.0242 ±0.0013	0.0580 ±0.0121	0.0223 ±0.0003	0.0363 ±0.0008	0.0106 ±0.0018	0.0265 ±0.0004
	sigmoid	0.0108 ±0.0006	0.0337 ±0.0018	0.0386 ±0.0055	0.0344 ±0.0013	0.0194 ±0.0026	0.0193 ±0.0016	0.0094 ±0.0004	0.0318 ±0.0055
	softplus	0.0072 ±0.0003	0.0145 ±0.0007	0.0217 ±0.0021	0.0252 ±0.0014	0.0163 ±0.0003	0.0195 ±0.0004	0.0068 ±0.0008	0.0402 ±0.0050
	LeakyReLU	0.0194 ±0.0008	0.0363 ±0.0016	0.0463 ±0.0014	0.0494 ±0.0026	0.0242 ±0.0013	0.0279 ±0.0008	0.0109 ±0.0004	0.0257 ±0.0008

G. Performance evaluation of PIG'N'PI with different activation functions for pairwise potential energy prediction. Table S9 reports the performance of PIG'N'PI with different activation functions to learn pairwise potential energy.

Table S9. Performance evaluation of PIG'N'PI with different activation functions for pairwise potential energy prediction. Results averaged across five experiments.

		Spring dim=2	Spring dim=3	Charge dim=2	Charge dim=3	Orbital dim=2	Orbital dim=3	Discnt dim=2	Discnt dim=3
MAE_{acc}	SiLU	0.0076 ±0.0003	0.0099 ±0.0007	0.0225 ±0.0012	0.1088 ±0.0079	0.0090 ±0.0004	0.0091 ±0.0004	0.0089 ±0.0002	0.0150 ±0.0022
	ReLU	3.2521 ±0.0818	5.0524 ±0.0796	6.2996 ±0.0043	1.8127 ±0.0017	5.6495 ±0.0788	3.8274 ±0.0224	1.5069 ±0.0501	2.8088 ±0.0224
	GELU	0.0063 ±0.0004	0.0054 ±0.0003	0.0298 ±0.0010	0.1586 ±0.0061	0.0089 ±0.0001	0.0098 ±0.0004	0.0104 ±0.0009	0.0154 ±0.0005
	tanh	0.0223 ±0.0023	0.0366 ±0.0054	0.0499 ±0.0016	0.1949 ±0.0112	0.0139 ±0.0001	0.0187 ±0.0006	0.0330 ±0.0010	0.0889 ±0.0104
	sigmoid	0.0949 ±0.0915	0.0432 ±0.0038	0.0631 ±0.0029	0.1022 ±0.0092	0.0206 ±0.0012	0.0290 ±0.0015	0.0299 ±0.0014	0.0638 ±0.0026
	softplus	0.0259 ±0.0029	0.0271 ±0.0019	0.0516 ±0.0034	0.0870 ±0.0049	0.0113 ±0.0005	0.0161 ±0.0018	0.0274 ±0.0023	0.0430 ±0.0021
	LeakyReLU	3.3248 ±0.0626	5.0494 ±0.0265	6.2987 ±0.0070	1.8135 ±0.0017	5.6056 ±0.0270	3.8106 ±0.0647	1.5031 ±0.0369	2.7777 ±0.0408
MAE_{ef}	SiLU	0.0023 ±0.0001	0.0037 ±0.0003	0.0080 ±0.0006	0.0223 ±0.0013	0.0058 ±0.0011	0.0053 ±0.0005	0.0016 ±3.4E-5	0.0030 ±0.0004
	ReLU	1.1132 ±0.0243	1.5625 ±0.0287	1.2551 ±0.0027	0.3854 ±0.0007	1.9323 ±0.0265	1.3612 ±0.0129	0.5471 ±0.0178	0.9503 ±0.0067
	GELU	0.0020 ±0.0001	0.0029 ±0.0007	0.0114 ±0.0001	0.0312 ±0.0016	0.0054 ±0.0008	0.0062 ±0.0004	0.0019 ±0.0002	0.0032 ±0.0001
	tanh	0.0081 ±0.0011	0.0136 ±0.0014	0.0214 ±0.0006	0.0719 ±0.0016	0.0109 ±0.0015	0.0150 ±0.0011	0.0069 ±0.0002	0.0223 ±0.0027
	sigmoid	0.0267 ±0.0290	0.0123 ±0.0010	0.0210 ±0.0013	0.0292 ±0.0026	0.0113 ±0.0007	0.0139 ±0.0009	0.0058 ±0.0003	0.0143 ±0.0006
	softplus	0.0068 ±0.0007	0.0103 ±0.0009	0.0202 ±0.0023	0.0260 ±0.0022	0.0073 ±0.0012	0.0084 ±0.0009	0.0054 ±0.0005	0.0097 ±0.0006
	LeakyReLU	1.1325 ±0.0143	1.5550 ±0.0120	1.2511 ±0.0026	0.3857 ±0.0011	1.9077 ±0.0125	1.3596 ±0.0250	0.5444 ±0.0174	0.9422 ±0.0112
MAE_{nf}	SiLU	0.0080 ±0.0003	0.0104 ±0.0007	0.0261 ±0.0014	0.1212 ±0.0085	0.0115 ±0.0006	0.0118 ±0.0005	0.0098 ±0.0002	0.0160 ±0.0023
	ReLU	3.3210 ±0.0732	5.1395 ±0.0846	7.1663 ±0.0054	1.9481 ±0.0020	7.2113 ±0.0851	4.9279 ±0.0347	1.5787 ±0.0533	2.8956 ±0.0199
	GELU	0.0067 ±0.0005	0.0059 ±0.0003	0.0347 ±0.0011	0.1757 ±0.0071	0.0114 ±0.0003	0.0128 ±0.0006	0.0111 ±0.0009	0.0162 ±0.0004
	tanh	0.0235 ±0.0023	0.0392 ±0.0056	0.0578 ±0.0018	0.2202 ±0.0131	0.0176 ±0.0003	0.0236 ±0.0007	0.0355 ±0.0010	0.0944 ±0.0110
	sigmoid	0.0993 ±0.0957	0.0447 ±0.0038	0.0738 ±0.0034	0.1137 ±0.0095	0.0268 ±0.0016	0.0369 ±0.0017	0.0320 ±0.0015	0.0672 ±0.0029
	softplus	0.0268 ±0.0030	0.0283 ±0.0019	0.0599 ±0.0037	0.0964 ±0.0059	0.0148 ±0.0006	0.0206 ±0.0019	0.0292 ±0.0023	0.0454 ±0.0025
	LeakyReLU	3.3932 ±0.0640	5.1328 ±0.0205	7.1647 ±0.0082	1.9487 ±0.0022	7.1476 ±0.0364	4.8920 ±0.0795	1.5798 ±0.0339	2.8733 ±0.0387
$MAE_{\triangle ep}$	SiLU	0.0005 ±1.4E-5	0.0016 ±0.0003	0.0096 ±0.0009	0.0156 ±0.0015	0.0048 ±0.0017	0.0031 ±0.0006	0.2197 ±0.0001	0.2344 ±0.0001
	ReLU	1.8798 ±0.2632	5.8125 ±0.2222	0.4592 ±0.0115	0.2061 ±0.0042	1.3700 ±0.1386	1.0480 ±0.0589	0.6844 ±0.0786	1.4246 ±0.1339
	GELU	0.0006 ±0.0001	0.0017 ±0.0007	0.0145 ±0.0007	0.0156 ±0.0020	0.0034 ±0.0011	0.0037 ±0.0006	0.2197 ±0.0001	0.2344 ±0.0001
	tanh	0.0030 ±0.0013	0.0037 ±0.0008	0.0312 ±0.0008	0.0803 ±0.0040	0.0080 ±0.0019	0.0102 ±0.0010	0.2202 ±0.0001	0.2353 ±0.0002

(Continued on next page)

Table S9. Continued: Performance evaluation of PIG'N'PI with different activation functions for pairwise potential energy prediction.

	sigmoid	0.0068 ±0.0094	0.0025 ±0.0002	0.0271 ±0.0033	0.0298 ±0.0048	0.0068 ±0.0012	0.0074 ±0.0014	0.2199 ±0.0002	0.2349 ±0.0005
	softplus	0.0015 ±0.0005	0.0080 ±0.0016	0.0315 ±0.0059	0.0369 ±0.0041	0.0079 ±0.0016	0.0065 ±0.0013	0.2199 ±0.0001	0.2349 ±0.0002
	LeakyReLU	2.0263 ±0.3663	5.2505 ±0.6543	0.4646 ±0.0083	0.2081 ±0.0034	1.5073 ±0.1474	1.1100 ±0.0630	0.7408 ±0.1149	1.3386 ±0.1336
$\text{MAE}_{\Delta\text{np}}$	SiLU	0.0016 ±0.0001	0.0062 ±0.0012	0.0179 ±0.0011	0.0381 ±0.0037	0.0129 ±0.0046	0.0074 ±0.0009	0.9319 ±0.0005	0.9322 ±0.0004
	ReLU	8.6263 ±1.4047	22.7924 ±2.6079	1.5514 ±0.0545	0.6727 ±0.0154	5.3176 ±1.0821	4.0300 ±0.2800	3.2903 ±0.4927	6.6232 ±1.1136
	GELU	0.0015 ±0.0002	0.0044 ±0.0020	0.0263 ±0.0013	0.0441 ±0.0053	0.0085 ±0.0026	0.0099 ±0.0020	0.9322 ±0.0003	0.9319 ±0.0002
	tanh	0.0068 ±0.0016	0.0097 ±0.0009	0.0469 ±0.0023	0.1859 ±0.0073	0.0173 ±0.0019	0.0234 ±0.0029	0.9338 ±0.0009	0.9338 ±0.0014
	sigmoid	0.0220 ±0.0312	0.0076 ±0.0007	0.0398 ±0.0040	0.0689 ±0.0111	0.0203 ±0.0044	0.0211 ±0.0024	0.9324 ±0.0011	0.9331 ±0.0017
	softplus	0.0051 ±0.0017	0.0339 ±0.0095	0.0463 ±0.0069	0.0794 ±0.0108	0.0331 ±0.0107	0.0307 ±0.0097	0.9323 ±0.0005	0.9330 ±0.0012
	LeakyReLU	10.3190 ±2.5713	22.1011 ±4.2016	1.5917 ±0.0667	0.6743 ±0.0250	6.1927 ±1.0489	4.2487 ±0.3877	3.4278 ±1.0505	6.1117 ±1.1812
$\text{MAE}^{P}_{\text{symm}}$	SiLU	0.0007 ±0.0001	0.0025 ±0.0008	0.0074 ±0.0010	0.0062 ±0.0005	0.0252 ±0.0065	0.0422 ±0.0144	0.0003 ±2.1E-5	0.0005 ±2.6E-5
	ReLU	1.8823 ±0.4517	3.6725 ±0.8805	0.1467 ±0.0339	0.0702 ±0.0076	0.9496 ±0.0576	0.7473 ±0.0270	0.5693 ±0.1220	1.1960 ±0.2500
	GELU	0.0009 ±0.0005	0.0047 ±0.0022	0.0090 ±0.0005	0.0078 ±0.0009	0.0521 ±0.0149	0.0460 ±0.0176	0.0004 ±0.0000	0.0008 ±0.0000
	tanh	0.0202 ±0.0327	0.0056 ±0.0022	0.0155 ±0.0019	0.0215 ±0.0007	0.1968 ±0.0592	0.1172 ±0.0242	0.0010 ±0.0001	0.0024 ±0.0001
	sigmoid	0.0059 ±0.0076	0.0028 ±0.0003	0.0237 ±0.0071	0.0130 ±0.0010	0.0294 ±0.0127	0.0751 ±0.0181	0.0006 ±0.0000	0.0017 ±0.0001
	softplus	0.0017 ±0.0007	0.0112 ±0.0041	0.0151 ±0.0070	0.0115 ±0.0012	0.0481 ±0.0427	0.0328 ±0.0257	0.0007 ±0.0001	0.0015 ±0.0001
	LeakyReLU	2.1660 ±0.5849	3.2254 ±1.0211	0.1492 ±0.0203	0.0688 ±0.0052	0.9544 ±0.0521	0.7962 ±0.0972	0.5670 ±0.2456	0.9258 ±0.1685

(The end)

H. Imposing symmetry regularization on the baseline model to learn force. Table S10 reports the performance of the baseline model with symmetry regularization (see the discussion in Sec. E and Sec. C). Results show that such symmetry regularization improves the performance of the baseline model with respect to $\mathsf{MAE}^{F}_{\mathsf{symm}}$, which was expected since the symmetry term was minimized. Furthermore, the symmetry regularization makes the baseline model perform better in terms of $\mathsf{MAE}_{\mathsf{acc}}$, $\mathsf{MAE}_{\mathsf{ef}}$ and $\mathsf{MAE}_{\mathsf{nf}}$ on several datasets. However, PIG'N'PI still significantly outperforms the extended baseline in terms of $\mathsf{MAE}_{\mathsf{acc}}$, $\mathsf{MAE}_{\mathsf{ef}}$ and $\mathsf{MAE}_{\mathsf{nf}}$, which are the most relevant performance evaluation metrics for physics-consistent particle interactions.

Table S10. Comparison of pairwise force prediction of the baseline model, extended baseline model with symmetry regularization with different weights and PIG'N'PI. Results averaged across five experiments.

		Spring dim=2	Spring dim=3	Charge dim=2	Charge dim=3	Orbital dim=2	Orbital dim=3	Discnt dim=2	Discnt dim=3
$\mathsf{MAE}_{\mathsf{acc}}$	Baseline	0.0565 ±0.0023	0.1076 ±0.0012	0.2521 ±0.0173	0.3824 ±0.0559	0.0437 ±0.0026	0.0439 ±0.0014	0.0592 ±0.0015	0.1171 ±0.0010
	$\alpha = 0.1$	0.0756 ±0.0015	0.1390 ±0.0019	0.2611 ±0.0279	0.2864 ±0.0117	0.0544 ±0.0017	0.0567 ±0.0007	0.0623 ±0.0016	0.1256 ±0.0020
	$\alpha = 1.0$	0.0743 ±0.0026	0.1465 ±0.0013	0.2431 ±0.0092	0.3121 ±0.0436	0.0799 ±0.0022	0.0769 ±0.0014	0.0571 ±0.0027	0.1135 ±0.0026
	$\alpha = 10$	0.0676 ±0.0012	0.1214 ±0.0018	5.4372 ±0.1597	0.7785 ±0.0027	0.0769 ±0.0037	0.0740 ±0.0012	0.0538 ±0.0019	0.1017 ±0.0039
	$\alpha = 100$	0.0770 ±0.0027	0.1381 ±0.0024	5.5282 ±0.0249	0.7758 ±0.0042	0.0902 ±0.0013	0.1068 ±0.0028	0.0573 ±0.0022	0.1064 ±0.0017
	PIG'N'PI	**0.0206 ±0.0009**	**0.0278 ±0.0021**	**0.0425 ±0.0053**	**0.1191 ±0.0027**	**0.0202 ±0.0003**	**0.0182 ±0.0003**	**0.0227 ±0.0019**	**0.0399 ±0.0011**
$\mathsf{MAE}_{\mathsf{ef}}$	Baseline	2.3979 ±0.2095	3.8952 ±0.7178	1.1832 ±0.0955	0.6447 ±0.1118	4.1010 ±0.1467	3.5379 ±0.7571	1.6536 ±0.0640	2.5803 ±0.2886
	$\alpha = 0.1$	1.6465 ±0.1523	2.6250 ±0.1347	1.2490 ±0.1001	0.3751 ±0.0167	2.4493 ±0.1253	1.7196 ±0.1193	0.5302 ±0.0191	1.2120 ±0.0456
	$\alpha = 1.0$	1.5304 ±0.1035	2.4136 ±0.0925	1.2811 ±0.0343	0.3754 ±0.0043	2.3558 ±0.1536	1.7502 ±0.0739	0.5250 ±0.0861	0.9572 ±0.0394
	$\alpha = 10$	1.6178 ±0.0424	2.3723 ±0.0327	1.2531 ±0.0004	0.3790 ±1E-11	2.3495 ±0.0267	1.7543 ±0.0317	0.4819 ±0.0125	0.9746 ±0.0191
	$\alpha = 100$	1.5694 ±0.0167	2.3943 ±0.0078	1.2528 ±2E-11	0.3790 ±9E-12	2.3632 ±0.0077	1.7505 ±0.0066	0.4893 ±0.0053	0.9796 ±0.0056
	PIG'N'PI	**0.0063 ±0.0002**	**0.0101 ±0.0007**	**0.0136 ±0.0023**	**0.0363 ±0.0015**	**0.0093 ±0.0002**	**0.0095 ±0.0001**	**0.0040 ±0.0004**	**0.0079 ±0.0002**
$\mathsf{MAE}_{\mathsf{nf}}$	Baseline	11.652 ±0.9890	20.967 ±3.8552	6.831 ±0.5548	3.804 ±0.7523	18.194 ±0.6884	16.677 ±3.5212	10.786 ±0.3764	15.651 ±1.7983
	$\alpha = 0.1$	7.9466 ±0.7318	14.104 ±0.7268	7.2125 ±0.5854	1.9825 ±0.0927	10.675 ±0.5594	7.9656 ±0.5602	3.4844 ±0.1278	7.4980 ±0.2871
	$\alpha = 1.0$	7.3867 ±0.5031	12.954 ±0.4993	7.3978 ±0.2000	1.9827 ±0.0248	10.257 ±0.6695	8.1068 ±0.3377	3.4632 ±0.5716	5.9257 ±0.2496
	$\alpha = 10$	7.8101 ±0.2030	12.734 ±0.1777	7.2335 ±0.0002	2.0040 ±4.4E-8	10.228 ±0.1156	8.1252 ±0.1463	3.1834 ±0.0838	6.0471 ±0.1201
	$\alpha = 100$	7.5755 ±0.0808	12.851 ±0.0429	7.2335 ±1.3E-7	2.0040 ±2.5E-8	10.289 ±0.0334	8.1077 ±0.0307	3.2333 ±0.0349	6.0803 ±0.0346
	PIG'N'PI	**0.0219 ±0.0010**	**0.0292 ±0.0022**	**0.0488 ±0.0059**	**0.1317 ±0.0033**	**0.0260 ±0.0005**	**0.0233 ±0.0004**	**0.0239 ±0.0020**	**0.0419 ±0.0011**
$\mathsf{MAE}^{F}_{\mathsf{symm}}$	Baseline	1.1099 ±0.0785	1.7452 ±0.0467	0.1248 ±0.0137	0.6938 ±0.2670	2.2074 ±0.1852	1.7684 ±0.0941	0.9399 ±0.0257	1.4118 ±0.0722
	$\alpha = 0.1$	0.1116 ±0.0057	0.2262 ±0.0107	0.0718 ±0.0057	0.0243 ±0.0005	0.1979 ±0.0024	0.1863 ±0.0009	0.0418 ±0.0046	0.0988 ±0.0069
	$\alpha = 1.0$	0.0057 ±0.0003	0.0129 ±0.0004	0.0160 ±0.0005	0.0066 ±0.0014	0.0084 ±0.0003	0.0083 ±0.0002	0.0039 ±0.0004	0.0076 ±0.0002
	$\alpha = 10$	0.0012 ±3.0E-5	0.0023 ±0.0001	0.0010 ±0.0014	1.2E-6 ±1.0E-6	0.0013 ±0.0001	0.0018 ±0.0001	0.0008 ±2.9E-5	0.0016 ±0.0001
	$\alpha = 100$	0.0002 ±8.3E-6	0.0004 ±1.9E-5	2.4E-6 ±2.1E-6	6.7E-7 ±6.9E-7	0.0003 ±7.6E-6	0.0004 ±1.4E-5	0.0002 ±1.6E-5	0.0003 ±1.3E-5
	PIG'N'PI	**0.0075 ±0.0003**	**0.0133 ±0.0008**	**0.0185 ±0.0036**	**0.0345 ±0.0017**	**0.0136 ±0.0004**	**0.0134 ±0.0004**	**0.0026 ±0.0004**	**0.0066 ±0.0001**

I. Robustness to noise. In this subsection, we evaluate the performance of the ML models under the assumption that the position measurements are impacted by noise. To simulate measurement noise, we impose white noise on the particle positions at each time step. Then, we compute particle velocities and accelerations from the noisy positions. Here, we consider the following equation to impose noise on the measured positions:

$$\tilde{r}_{i,k}^{t} \leftarrow r_{i,k}^{t} + \beta \times X_{i,k}^{t} \qquad [11]$$

where $\tilde{r}_{i,k}^{t}$ is the k-th dimension of the noisy position of particle i at time t, $X_{i,k}^{t} \sim \mathcal{N}(0,\, 1)$ is the random number sampled independently from the standard normal distribution and β is a constant controlling the level of noise. The second term in Eq. (11) represents the noise that is relevant to how we measure the position and how we discretize the space.

Different values for β will result in different noise levels for both inputs (position and velocity) and the learning target (acceleration). Here, we define the noise level as the **average relative change of the target**:

$$\text{noise level} = \frac{1}{T}\frac{1}{|V|}\frac{1}{d}\sum_{t=1}^{T}\sum_{i\in V}\sum_{k\in d}\frac{|\tilde{a}_{i,k}^{t} - a_{i,k}^{t}|}{|a_{i,k}^{t}|} \qquad [12]$$

Here, $\tilde{a}_{i,k}^{t}$ is the k-th dimension of the noisy acceleration of particle i at time t*. We test 1e-7, 5e-7, 1e-6, 5e-6 and 1e-5 as the values for β. The corresponding noise levels of each dataset are summarized in Table S11.

Table S11. The noise level (Eq. (12)) of each dataset with different values for β.

	Spring dim=2	Spring dim=3	Charge dim=2	Charge dim=3	Orbital dim=2	Orbital dim=3	Discnt dim=2	Discnt dim=3
β=1e-7	0.0117	0.0075	0.3057	1.6369	0.0036	0.0092	0.0102	0.0187
β=5e-7	0.0405	0.0347	1.6401	6.1344	0.0182	0.0399	0.0515	0.0696
β=1e-6	0.1509	0.0790	3.0269	18.979	0.0369	0.0786	0.0979	0.1284
β=5e-6	0.5137	0.3936	14.767	43.030	0.1696	0.3980	0.4634	0.9941
β=1e-5	0.8897	0.7108	29.881	119.34	0.3664	0.8667	0.9809	1.9767

Table S12 and Table S13 report the performances of baseline and PIG'N'PI to learn pairwise force with the noisy input.

The results show the performance of PIG'N'PI decreases with increasing noise level. This makes sense because adding noise makes the training target less similar to the uncorrupted target that is associated with the pairwise force (note that we do not corrupt the ground-truth pairwise forces during evaluation). However, PIG'N'PI can still preform reasonably well with small scale noise.

The performance of baseline model fluctuates significantly with different noise levels. This also makes sense because the baseline model does not learn the particle interactions.

Developing PIG'N'PI further to make it even more robust to noisy input is left for future work.

*When computing the noise level, we only consider those $|a_{i,k}^{t}|$ that are strictly larger than zero because we want to avoid dividing zero.

Table S12. Quality of pairwise force prediction of the baseline model with noisy data. The imposed noise corresponds to Eq. (11). "Uncorrupted" refers to the data without noise. Results averaged across five experiments.

		Spring dim=2	Spring dim=3	Charge dim=2	Charge dim=3	Orbital dim=2	Orbital dim=3	Discnt dim=2	Discnt dim=3
MAE_{acc}	Uncorrupted	0.0565 ±0.0023	0.1076 ±0.0012	0.2521 ±0.0173	0.3824 ±0.0559	0.0437 ±0.0026	0.0439 ±0.0014	0.0592 ±0.0015	0.1171 ±0.0010
	β=1e-7	0.0586 ±0.0016	0.1085 ±0.0023	0.2753 ±0.0194	0.3544 ±0.0220	0.0435 ±0.0006	0.0446 ±0.0009	0.0586 ±0.0013	0.1158 ±0.0034
	β=5e-7	0.0639 ±0.0037	0.1165 ±0.0027	0.2735 ±0.0246	0.4071 ±0.0286	0.0497 ±0.0014	0.0571 ±0.0010	0.0719 ±0.0028	0.1317 ±0.0046
	β=1e-6	0.0767 ±0.0012	0.1340 ±0.0013	0.2933 ±0.0159	0.3973 ±0.0391	0.0654 ±0.0006	0.0828 ±0.0008	0.0890 ±0.0013	0.1556 ±0.0031
	β=5e-6	0.2430 ±0.0011	0.3758 ±0.0016	0.4138 ±0.0198	0.6577 ±0.0276	0.2228 ±0.0014	0.3331 ±0.0013	0.2553 ±0.0032	0.4174 ±0.0016
	β=1e-5	0.4694 ±0.0039	0.7196 ±0.0021	0.6222 ±0.0159	1.0038 ±0.0572	0.4370 ±0.0011	0.6683 ±0.0020	0.4809 ±0.0020	0.7552 ±0.0040
MAE_{ef}	Uncorrupted	2.3979 ±0.2095	3.8952 ±0.7178	1.1832 ±0.0955	0.6447 ±0.1118	4.1010 ±0.1467	3.5379 ±0.7571	1.6536 ±0.0640	2.5803 ±0.2886
	β=1e-7	1.9321 ±0.6308	4.2515 ±0.4175	1.3544 ±0.0752	0.5249 ±0.0468	3.3631 ±0.7493	3.8075 ±0.1573	1.6651 ±0.2816	2.6424 ±0.2322
	β=5e-7	2.5900 ±0.4020	4.0157 ±0.3880	1.2892 ±0.0139	0.6376 ±0.0887	2.5351 ±0.8932	3.5454 ±0.4215	1.6866 ±0.1694	1.9837 ±0.2123
	β=1e-6	2.6234 ±0.9036	3.8559 ±0.4356	1.3248 ±0.0811	0.5286 ±0.0782	4.0880 ±1.3048	3.7811 ±0.3673	1.3008 ±0.2183	2.3136 ±0.4424
	β=5e-6	1.6493 ±0.8436	3.9151 ±0.5906	1.3440 ±0.1431	0.6125 ±0.0889	3.9054 ±1.4067	3.2323 ±0.6269	1.3307 ±0.1672	2.0825 ±0.3995
	β=1e-5	2.2223 ±0.4609	3.3053 ±0.4277	1.2780 ±0.1208	0.6939 ±0.1686	4.4303 ±0.9807	2.9186 ±0.5783	1.2169 ±0.3209	1.9050 ±0.3288
MAE_{nf}	Uncorrupted	11.652 ±0.9890	20.967 ±3.8552	6.8310 ±0.5548	3.8038 ±0.7523	18.194 ±0.6884	16.677 ±3.5212	10.786 ±0.3764	15.651 ±1.7983
	β=1e-7	9.3805 ±3.0398	22.903 ±2.3024	7.8383 ±0.4344	3.0275 ±0.2899	14.940 ±3.3092	17.918 ±0.7365	10.838 ±1.8261	15.971 ±1.4671
	β=5e-7	12.588 ±1.9510	21.635 ±2.0799	7.455 ±0.0881	3.751 ±0.5713	11.284 ±3.9460	16.671 ±1.9563	10.957 ±1.0616	11.936 ±1.3494
	β=1e-6	12.746 ±4.3777	20.718 ±2.3664	7.661 ±0.4736	2.987 ±0.5201	18.104 ±5.7265	17.759 ±1.7564	8.3936 ±1.4703	14.016 ±2.7942
	β=5e-6	7.9912 ±4.0598	21.037 ±3.1630	7.8270 ±0.8340	3.6208 ±0.5847	17.303 ±6.1886	15.218 ±2.9087	8.4946 ±1.0542	12.392 ±2.4643
	β=1e-5	10.798 ±2.2379	17.752 ±2.3123	7.5451 ±0.7052	4.2474 ±1.0325	19.595 ±4.3285	13.720 ±2.6955	7.6793 ±2.0393	11.189 ±2.0076
MAE^F_{symm}	Uncorrupted	1.1099 ±0.0785	1.7452 ±0.0467	0.1248 ±0.0137	0.6938 ±0.2670	2.2074 ±0.1852	1.7684 ±0.0941	0.9399 ±0.0257	1.4118 ±0.0722
	β=1e-7	1.0733 ±0.1043	1.8067 ±0.0760	0.1232 ±0.0352	0.4565 ±0.0538	1.9961 ±0.1710	1.9239 ±0.1455	1.0063 ±0.1074	1.4453 ±0.0999
	β=5e-7	0.9284 ±0.0798	1.8013 ±0.0401	0.1666 ±0.0190	0.6596 ±0.1836	2.0250 ±0.1278	1.7911 ±0.0679	0.9921 ±0.1084	1.3530 ±0.0336
	β=1e-6	1.0539 ±0.0690	1.6816 ±0.0809	0.1592 ±0.0224	0.3622 ±0.2052	2.1475 ±0.1492	1.7512 ±0.1160	0.9143 ±0.1050	1.3089 ±0.1026
	β=5e-6	0.8486 ±0.0614	1.6024 ±0.0432	0.1551 ±0.0126	0.5979 ±0.2170	1.9721 ±0.1302	1.7956 ±0.0726	0.8552 ±0.1154	1.1735 ±0.1105
	β=1e-5	0.7940 ±0.1133	1.4555 ±0.0619	0.1581 ±0.0241	0.7711 ±0.3974	2.0862 ±0.1608	1.5985 ±0.0812	0.7857 ±0.1024	1.1565 ±0.0908

Table S13. Quality of pairwise force prediction of the PIG'N'PI with noisy data. The imposed noise corresponds to Eq. (11). "Uncorrupted" refers to the data without noise. Results averaged across five experiments.

		Spring dim=2	Spring dim=3	Charge dim=2	Charge dim=3	Orbital dim=2	Orbital dim=3	Discnt dim=2	Discnt dim=3
MAE_{acc}	Uncorrupted	0.0206 ±0.0009	0.0278 ±0.0021	0.0425 ±0.0053	0.1191 ±0.0027	0.0202 ±0.0003	0.0182 ±0.0003	0.0227 ±0.0019	0.0399 ±0.0011
	β=1e-7	0.0213 ±0.0009	0.0305 ±0.0020	0.0421 ±0.0031	0.1208 ±0.0030	0.0208 ±0.0005	0.0203 ±0.0005	0.0274 ±0.0045	0.0429 ±0.0009
	β=5e-7	0.0315 ±0.0007	0.0449 ±0.0012	0.0510 ±0.0020	0.1393 ±0.0055	0.0302 ±0.0004	0.0374 ±0.0003	0.0377 ±0.0008	0.0614 ±0.0006
	β=1e-6	0.0499 ±0.0004	0.0720 ±0.0007	0.0697 ±0.0021	0.1704 ±0.0038	0.0473 ±0.0005	0.0658 ±0.0001	0.0585 ±0.0038	0.0902 ±0.0012
	β=5e-6	0.2050 ±0.0004	0.3062 ±0.0004	0.2209 ±0.0032	0.4163 ±0.0063	0.2033 ±0.0003	0.3088 ±0.0010	0.2124 ±0.0027	0.3257 ±0.0006
	β=1e-5	0.4060 ±0.0009	0.6136 ±0.0008	0.4215 ±0.0016	0.7661 ±0.0097	0.4102 ±0.0009	0.6348 ±0.0026	0.4146 ±0.0017	0.6231 ±0.0007
MAE_{ef}	Uncorrupted	0.0063 ±0.0002	0.0101 ±0.0007	0.0136 ±0.0023	0.0363 ±0.0015	0.0093 ±0.0002	0.0095 ±0.0001	0.0040 ±0.0004	0.0079 ±0.0002
	β=1e-7	0.0064 ±0.0002	0.0107 ±0.0007	0.0135 ±0.0016	0.0359 ±0.0009	0.0092 ±0.0003	0.0101 ±0.0003	0.0047 ±0.0010	0.0082 ±0.0001
	β=5e-7	0.0068 ±0.0002	0.0112 ±0.0005	0.0143 ±0.0011	0.0381 ±0.0016	0.0097 ±0.0001	0.0111 ±0.0001	0.0043 ±0.0002	0.0089 ±0.0001
	β=1e-6	0.0078 ±0.0001	0.0131 ±0.0004	0.0163 ±0.0013	0.0413 ±0.0016	0.0108 ±0.0002	0.0136 ±0.0002	0.0055 ±0.0013	0.0106 ±0.0003
	β=5e-6	0.0162 ±0.0002	0.0275 ±0.0004	0.0265 ±0.0024	0.0572 ±0.0024	0.0223 ±0.0005	0.0373 ±0.0010	0.0114 ±0.0027	0.0211 ±0.0006
	β=1e-5	0.0321 ±0.0007	0.0545 ±0.0006	0.0411 ±0.0005	0.0984 ±0.0065	0.0421 ±0.0004	0.0826 ±0.0029	0.0247 ±0.0031	0.0410 ±0.0007
MAE_{nf}	Uncorrupted	0.0219 ±0.0010	0.0292 ±0.0022	0.0488 ±0.0059	0.1317 ±0.0033	0.0260 ±0.0005	0.0233 ±0.0004	0.0239 ±0.0020	0.0419 ±0.0011
	β=1e-7	0.0230 ±0.0010	0.0324 ±0.0021	0.0486 ±0.0035	0.1334 ±0.0034	0.0266 ±0.0007	0.0260 ±0.0006	0.0294 ±0.0046	0.0456 ±0.0008
	β=5e-7	0.0370 ±0.0007	0.0528 ±0.0011	0.0607 ±0.0024	0.1581 ±0.0058	0.0386 ±0.0005	0.0481 ±0.0004	0.0439 ±0.0008	0.0704 ±0.0007
	β=1e-6	0.0610 ±0.0004	0.0880 ±0.0007	0.0846 ±0.0023	0.1974 ±0.0046	0.0606 ±0.0007	0.0846 ±0.0002	0.0701 ±0.0038	0.1070 ±0.0011
	β=5e-6	0.2605 ±0.0003	0.3876 ±0.0004	0.2782 ±0.0036	0.5092 ±0.0082	0.2602 ±0.0006	0.3968 ±0.0011	0.2673 ±0.0027	0.4078 ±0.0005
	β=1e-5	0.5143 ±0.0008	0.7800 ±0.0008	0.5353 ±0.0019	0.9458 ±0.0102	0.5273 ±0.0009	0.8101 ±0.0029	0.5250 ±0.0019	0.7843 ±0.0006
MAE^{F}_{symm}	Uncorrupted	0.0075 ±0.0003	0.0133 ±0.0008	0.0185 ±0.0036	0.0345 ±0.0017	0.0136 ±0.0004	0.0134 ±0.0004	0.0026 ±0.0004	0.0066 ±0.0001
	β=1e-7	0.0076 ±0.0002	0.0139 ±0.0008	0.0180 ±0.0030	0.0339 ±0.0010	0.0132 ±0.0005	0.0141 ±0.0005	0.0031 ±0.0006	0.0069 ±0.0000
	β=5e-7	0.0083 ±0.0003	0.0149 ±0.0007	0.0201 ±0.0022	0.0369 ±0.0016	0.0139 ±0.0002	0.0155 ±0.0002	0.0032 ±0.0002	0.0079 ±0.0002
	β=1e-6	0.0098 ±0.0001	0.0173 ±0.0005	0.0223 ±0.0017	0.0407 ±0.0012	0.0154 ±0.0002	0.0194 ±0.0005	0.0046 ±0.0012	0.0096 ±0.0001
	β=5e-6	0.0222 ±0.0003	0.0382 ±0.0004	0.0354 ±0.0023	0.0653 ±0.0023	0.0314 ±0.0008	0.0529 ±0.0014	0.0124 ±0.0037	0.0238 ±0.0011
	β=1e-5	0.0450 ±0.0010	0.0768 ±0.0011	0.0485 ±0.0016	0.1184 ±0.0085	0.0601 ±0.0008	0.1174 ±0.0049	0.0314 ±0.0048	0.0518 ±0.0011

J. Visualization of force and potential functions used in simulation. Fig. S2 shows the inter-particle potential energy P and the inter-particle pairwise force $\boldsymbol{F}$ used for generating the simulations. P and $\boldsymbol{F}$ are the functions of relative distance.

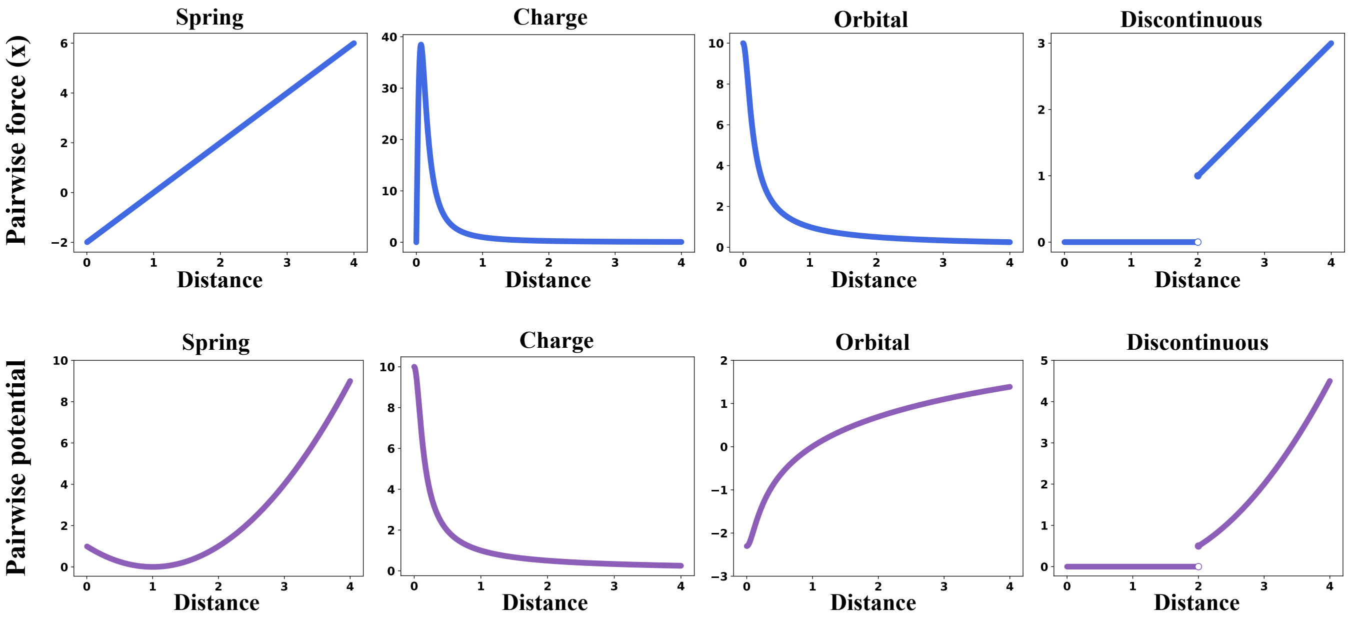

Fig. S2. Visualization of pairwise force and potential with different distances. Blue color shows the pairwise force in x dimension as the function of the relative distance between particles. Purple color in second row shows the pairwise potential with different distance. In this visualization, we set the electric charge and particle masses to one.

References

1. P Lemos, N Jeffrey, M Cranmer, P Battaglia, S Ho, Rediscovering newton's gravity and solar system properties using deep learning and inductive biases in *SimDL Workshop at ICLR*. (2021).
